# Integration of expression QTLs with fine mapping via SuSiE

**DOI:** 10.1371/journal.pgen.1010929

**Published:** 2024-01-25

**Authors:** Xiangyu Zhang, Wei Jiang, Hongyu Zhao

**Affiliations:** Department of Biostatistics, School of Public Health, Yale University, New Haven, Connecticut, United States of America; Stanford University, UNITED STATES

## Abstract

Genome-wide association studies (GWASs) have achieved remarkable success in associating thousands of genetic variants with complex traits. However, the presence of linkage disequilibrium (LD) makes it challenging to identify the causal variants. To address this critical gap from association to causation, many fine-mapping methods have been proposed to assign well-calibrated probabilities of causality to candidate variants, taking into account the underlying LD pattern. In this manuscript, we introduce a statistical framework that incorporates expression quantitative trait locus (eQTL) information to fine-mapping, built on the sum of single-effects (SuSiE) regression model. Our new method, SuSiE^2^, connects two SuSiE models, one for eQTL analysis and one for genetic fine-mapping. This is achieved by first computing the posterior inclusion probabilities (PIPs) from an eQTL-based SuSiE model with the expression level of the candidate gene as the phenotype. These calculated PIPs are then utilized as prior inclusion probabilities for risk variants in another SuSiE model for the trait of interest. By prioritizing functional variants within the candidate region using eQTL information, SuSiE^2^ improves SuSiE by increasing the detection rate of causal SNPs and reducing the average size of credible sets. We compared the performance of SuSiE^2^ with other multi-trait fine-mapping methods with respect to power, coverage, and precision through simulations and applications to the GWAS results of Alzheimer’s disease (AD) and body mass index (BMI). Our results demonstrate the better performance of SuSiE^2^, both when the in-sample linkage disequilibrium (LD) matrix and an external reference panel is used in inference.

## Author summary

Genome-wide association studies (GWASs) have proven powerful in detecting genetic variants associated with complex traits. However, there are challenges in distinguishing the causal variants from other variants strongly correlated with them. To better identify causal SNPs, many fine-mapping methods have been proposed to assign well-calibrated probabilities of causality to candidate variants. We introduce a statistical framework that incorporates expression quantitative trait locus (eQTL) information to fine-mapping, which can improve the accuracy and efficiency of association studies by prioritizing functional variants within the risk genes before evaluating the causation. Our new fine-mapping framework, SuSiE^2^, connects two sum of single-effects (SuSiE) models, one for eQTL analysis and one for genetic fine-mapping. The posterior inclusion probabilities from an eQTL-based SuSiE model are utilized as prior inclusion probabilities for risk variants in another SuSiE model for the trait of interest. Through simulations and real data analyses focused on body mass index and Alzheimer’s disease, we demonstrate that SuSiE^2^ improves fine-mapping results by increasing statistical power, having appropriate coverage, and reducing the average size of credible sets.

## Introduction

Over the past decades, genome-wide association studies (GWASs) have achieved remarkable success in detecting thousands of genetic variants that are associated with complex traits [1]. While GWASs have proven powerful in identifying genomic loci harboring causal variants, they encounter challenges in identifying the underlying causal variants. There is limited statistical power to distinguish causal variants from other variants in strong linkage disequilibrium (LD) through marginal association analysis [2, 3].

Genetic fine-mapping aims at inferring the causal genetic variants responsible for complex traits in a candidate region through disentangling LD patterns. Many fine-mapping methods have been devised to assign well-calibrated probabilities of causality to candidate variants, taking into account the underlying LD pattern. For instance, some methods in the early stage estimate the probability of causality for each SNP under the assumption that each risk locus only harbors one causal variant [4, 5]. To avoid this strict assumption, CAVIAR [6] estimates the posterior inclusion probability (PIP) of each variant as a causal factor by jointly modeling the observed association statistics among all risk variants. Because of the heavy computational burden, CAVIAR makes the assumption that the total number of causal SNPs in a region is bounded by at most six, which leads to a major limitation. Under a similar statistical model, FINEMAP [7] enhances the computational efficiency by replacing the exhaustive search algorithm in CAVIAR with a shotgun stochastic search. However, this method is still computationally intensive. SuSiE [8], on the other hand, introduces a novel approach to variable selection in linear regression problems, where genetic fine-mapping is an important application. Building upon Bayesian variable selection in regression (BVSR) [9], SuSiE develops an Iterative Bayesian Stepwise Selection (IBSS) algorithm to generate credible sets (CSs) that contain multiple highly correlated variables. The additive structure of the SuSiE model facilitates more accurate inference and improves computational efficiency, thereby enhancing the overall effectiveness of genetic fine-mapping.

In recent years, expression quantitative trait locus (eQTL) studies have revealed an abundance of quantitative trait loci (QTLs) for gene expression [10]. Integrating eQTL information into fine-mapping not only improves the accuracy and efficiency of association studies by prioritizing functional variants within the risk genes but also aids in understanding the mechanisms underlying a genetic risk locus [11, 12]. Generally, there are three approaches to incorporating eQTL signals into fine-mapping. The first approach involves conducting a colocalization analysis to determine whether the same variant is significant in both GWASs and eQTL studies. However, most colocalization methods, such as COLOC [13], eCAVIAR [14], and coloc-SuSiE [15], primarily focus on estimating the probability that a variant is causal in both GWASs and eQTL studies. This differs from our objective of identifying the causal variants associated with complex traits. The second approach incorporates gene expression levels as functional annotations and assigns functional priors to risk variants. Well established fine-mapping methods incorporating annotations include PAINTOR [16], PolyFun+SuSiE [17], DAP [11], and SparsePro [18]. However, a significant drawback of the majority of these methods is that they are designed with two distinct modeling stages that employ different model settings for estimating prior probabilities and conducting fine-mapping. This disjoint approach can result in potentially suboptimal performance [19]. The third approach involves a multi-trait fine-mapping framework, where the phenotypes and gene expression levels are treated as correlated traits. Examples of such methods include mvSuSiE [20], flashfm [21], and fastPAINTOR [22]. However, current multi-trait fine-mapping methods face limitations when integrating eQTLs into fine-mapping. For example, mvSuSiE assumes that each trait is measured in all samples, and fastPAINTOR makes the assumption that the same variants at the risk loci impact all traits. These assumptions likely do not hold for gene expression levels and the trait of interest.

In this study, we propose a new method of incorporating eQTL information to improve fine-mapping results based on the SuSiE framework. Our new method, named SuSiE^2^, begins by prioritizing risk variants using estimated PIPs from an eQTL-based SuSiE model with expression levels of risk genes serving as the phenotype. These PIPs are then utilized as prior inclusion probabilities in a standard SuSiE model for the trait of interest. Through simulations conducted on the UK Biobank (UKBB) samples, we demonstrate that SuSiE^2^ consistently improves the power of detecting causal SNPs compared with single-trait SuSiE and other multi-trait fine-mapping methods. SuSiE^2^ is also competitive in having the appropriate coverage and reducing the average size of CSs, whether using an in-sample LD matrix or an external reference panel. In real data analyses, SuSiE^2^ improves the performance of fine-mapping for body mass index (BMI) and identifies more Alzheimer’s disease (AD) associated SNPs predicted from single-cell epigenomic data.

## Materials and methods

### The sum of single effects regression model

To quantify the uncertainty in which variables should be selected, the BVSR methods calculate the marginal posterior inclusion probability (PIP) quantifying the probability that the variable is causal. The concept of PIP is widely adopted by fine-mapping methods for the selection of causal SNPs.

Traditional fine-mapping methods, such as CAVIAR and FINEMAP, are computationally intensive with complicated posterior distributions [7, 23]. The sum of single effects regression model (SuSiE) introduced by [8] takes advantage of the convenient analytic properties of a single-effect regression (SER) model [24]:

$$\begin{array}{c} \text{y}=\text{Xb}+\text{e},\;\text{e}∼N_{n}\left(0,\sigma^{2}I_{n}\right),\;\text{b}=\sum_{k=1}^{K}{\text{b}}_{k}, \end{array}$$

$$\begin{array}{c} {\text{b}}_{k}=\lambda_{k}{\text{c}}_{k},\;{\text{c}}_{k}∼Mult\left(1,\pi \right),\;\lambda_{k}∼N_{1}\left(0,\sigma_{0k}^{2}\right), \end{array}$$ (1)

where **y** is the *n*-vector of the response variable, **X** = (*x*_1_, …, *x*_*p*_) is a matrix containing *n* observations of *p* explanatory variables, **b**_1_, …, **b**_*K*_ represent the single-effect vectors each aiming to capture exactly one effect variable, *I*_*n*_ stands for the *n* × *n* identity matrix, *N*_*n*_ represents the *n*-variate normal distribution, $\sigma_{0}^{2}={\left(\sigma_{01}^{2},\ldots ,\sigma_{0K}^{2}\right)}^{T}$ are the prior variances of the non-zero effects which can be different for different **b**_*k*_, *K* is the assumed total number of effect variables, and ***π*** = (*π*_1_, …, *π*_*p*_)^*T*^ gives the prior probability of each variable being the effect variable.

To distinguish between different causal signals, SuSiE introduces the concept of a credible set (CS) of variables as below:

**Definition 1** *In a multiple-regression model, a level ρ credible set is defined to be a subset of variables that has probability ρ or greater of containing at least one effect variable*.

Different from existing BVSR models, SuSiE introduces a new model structure which naturally leads to an intuitive and fast iterative Bayesian stepwise selection (IBSS) algorithm [8] (S1 Text) for model fitting. Compared with traditional BVSR methods, SuSiE enjoys key advantages in interpretability of fine-mapping results and computational efficiency.

### Integrating eQTL information with fine-mapping

In the remaining parts of the method section, we will introduce a new framework to incorporate eQTL information into fine-mapping based on SuSiE.

Under the existence of strong LD, SuSiE assesses the uncertainty in variable selection by generating groups of variables, with each group aiming at capturing one effect variable. However, choosing the true causal variable from the credible set is still a difficult problem. One possible way to infer the effect variable more accurately is to integrate eQTL information into fine-mapping, as SNPs associated with complex traits are significantly more likely to be eQTLs [12]. Considering the effect of each risk variable on the gene expression level helps us to prioritize risk SNPs with the posterior probability of being the effect variable, which can replace the prior distribution used in the original SuSiE manuscript: ***π*** = (1/*p*, …, 1/*p*)^*T*^.

This new framework of eQTL-based fine-mapping study, named SuSiE^2^, connects two layers of SuSiE models for eQTL study and genetic fine-mapping, respectively. For the first layer, we use the gene expression levels as response variables and conduct a regression analysis on each risk gene region. This eQTL-based SuSiE model can be rewritten as (2):

$$\begin{array}{c} {\text{y}}^{e}={\text{Xb}}^{e}+{\text{e}}^{e},\;{\text{e}}^{e}∼N_{n_{e}}\left(0,{\left(\sigma^{e}\right)}^{2}I_{n_{e}}\right),\;{\text{b}}^{e}=\sum_{k=1}^{K^{e}}{\text{b}}_{k}^{e}, \end{array}$$

$$\begin{array}{c} {\text{b}}_{k}^{e}=\lambda_{k}^{e}{\text{c}}_{k}^{e},\;{\text{c}}_{k}^{e}∼Mult\left(1,\pi \right),\;\lambda_{k}^{e}∼N_{1}\left(0,{\left(\sigma_{0k}^{e}\right)}^{2}\right), \end{array}$$ (2)

where *n*_*e*_ is the eQTL study sample size, **y**^*e*^ is the *n*_*e*_-vector of gene expression levels, **b**^*e*^ is the *p*-vector of regression coefficients of risk variants for the gene expression, and ***π*** is the naive prior inclusion probability for the eQTL-based SuSiE. Assume that there are in total *K*^*e*^ causal signals for the gene expression level, we can output from (2) the PIPs for all the single effects, denoted as $\alpha_{1},\ldots \alpha_{K^{e}}$. The final PIPs for the eQTL study can be computed as:

$${\text{PIP}}^{e}=1-\prod_{k=1}^{K^{e}}\left(1-\alpha_{k}\right).$$

**PIP**^*e*^ represents the probability for each variant to be causal to the gene expression level. Under the assumption that trait-associated SNPs are more likely to be eQTLs, the PIPs from the eQTL-based SuSiE can serve as the prior distribution in the following SuSiE model for the trait of interest to highlight eQTLs in genetic fine-mapping:

$$\begin{array}{c} {\text{y}}^{t}={\text{Xb}}^{t}+{\text{e}}^{t},\;{\text{e}}^{t}∼N_{n_{t}}\left(0,{\left(\sigma^{t}\right)}^{2}I_{n_{t}}\right),\;{\text{b}}^{t}=\sum_{k=1}^{K^{t}}{\text{b}}_{k}^{t}, \end{array}$$

$$\begin{array}{c} {\text{b}}_{k}^{t}=\lambda_{k}^{t}{\text{c}}_{k}^{t},\;{\text{c}}_{k}^{t}∼Mult\left(1,{\text{PIP}}^{e}\right),\;\lambda_{k}^{t}∼N_{1}\left(0,{\left(\sigma_{0k}^{t}\right)}^{2}\right), \end{array}$$ (3)

where *n*_*t*_ is the sample size for the trait of interest, **y**^*t*^ is the *n*_*t*_-vector of the quantitative trait, **b**^*t*^ is the *p*-vector of regression coefficients of risk variants for this phenotype, and *K*^*t*^ is the total number of signals for the trait of interest.

Suppose from model (3) we detect single effects, with the corresponding PIPs denoted as $\beta_{1},\ldots \beta_{K^{t}}$, then the final PIPs for the trait of interest can be computed as:

$${\text{PIP}}^{t}=1-\prod_{k=1}^{K^{t}}\left(1-\beta_{k}\right),$$

which prioritizes the candidate variants for the trait of interest. From model (3) we can also obtain the variants contained in credible sets for the trait of interest after adjusting for the eQTL priors.

SuSiE^2^ provides an efficient method to increase the priority of eQTLs in the fine-mapping of the trait of interest. We also evaluated the potential impact of using the PIPs from the eQTL-based SuSiE (first layer) as prior for the trait-based SuSiE (second layer). Under the assumption that genetic variants influence the phenotype through gene expressions, we proved that the final PIPs (**PIP**^*t*^) from SuSiE^2^ are exactly the posterior probabilities for variants to be causal given both the phenotypes and gene expression data (see in S1 Text).

In the Materials and Methods section above, we describe the SuSiE model and the SuSiE^2^ framework based on individual-level genotype data. We note that SuSiE has been extended for summary statistics [25], which makes it competitive with other well-developed fine-mapping methods. Consequently, the SuSiE^2^ method is also applicable to analyzing summary statistics and a reference panel.

## Results

We demonstrate that integrating eQTL with fine-mapping via SuSiE^2^ can increase efficiency and accuracy through simulation studies and real data studies on BMI and AD. Compared with the original SuSiE, SuSiE^2^ can improve the results of fine-mapping in the following aspects while controlling the coverage rate of credible sets (CSs) at an appropriate level:

- SuSiE^2^ improves the likelihood of including causal variants in at least one CS.
- SuSiE^2^ improves the precision by reducing the average size for CSs.

### Simulation

We conducted simulations based on a two-layer linear regression model. Assuming a total of *L* risk genes associated with the trait of interest on this chromosome segment, we simulated the gene expression levels and the quantitative trait of interest through the following additive linear models:

$$\begin{array}{c} Y_{el}=\sum_{i=1}^{M_{el}}\beta_{eli}X_{i}+e_{l},\;e_{l}∼N\left(0,\sigma_{el}^{2}\right),\;\beta_{eli}∼N\left(0,\frac{1-\sigma_{el}^{2}}{M_{el}}\right),\;l=1,2,\ldots ,L, \end{array}$$

$$\begin{array}{c} Y_{t}=\sum_{i=1}^{M_{t}}\beta_{ti}X_{i}+\sum_{l=1}^{L}\gamma_{l}Y_{el}+e_{0},\;e_{0}∼N\left(0,\sigma_{t}^{2}\right),\;\beta_{ti}∼N\left(0,\frac{1-\sigma_{t}^{2}}{M_{t}}\right). \end{array}$$ (4)

Here, *Y*_*el*_ is the gene expression level for the *l*th risk gene, *Y*_*t*_ is the quantitative trait of interest, *M*_*el*_ represents the number of causal SNPs for the *l*th risk gene, *M*_*t*_ is the number of direct causal SNPs for the trait of interest, *X*_*i*_ is the standardized genotype for the *i*th SNP, *γ*_*l*_ is the coefficient for the expression level of the *l*th risk gene, and $\sigma_{el}^{2}$ and $\sigma_{t}^{2}$ are the variance of error terms for the *i*th gene expression level and trait of interest, respectively. The effect sizes of the causal SNPs were assumed to follow normal distributions with zero means and variances chosen to ensure *Var*(*Y*_*el*_) = *Var*(*Y*_*t*_) = 1. For each risk gene, half of the *M*_*el*_ causal SNPs were also contained in the *M*_*t*_ effect variants for *Y*_*t*_. Therefore, the causal SNPs can affect the trait of interest either directly or through their effects on gene expressions, or in both ways. Throughout our simulation, we fixed *γ*_*l*_ = 1.

#### Fine-mapping with the in-sample LD matrix

We simulated traits and gene expression levels based on model (4) under two scenarios:

(a) “All causal SNPs are eQTLs”: In this scenario, the number of risk genes (*L*) was 4, the number of causal SNPs for each risk gene (*M*_*el*_) was 2, and there were no causal SNPs outside of risk genes. Therefore, the total number of causal SNPs was 8. The proportion of variation explained by the SNPs (heritability) was selected from the set {0.02, 0.04, 0.06, 0.08, 0.1}.
(b) “Some causal SNPs are eQTLs”: This scenario involved four risk genes (*L* = 4), each with two causal SNPs for gene expression (*M*_*el*_ = 2). In addition, two causal SNPs were assumed outside of risk genes, bringing the total number of causal SNPs to 10. The heritability was chosen from the set {0.02, 0.04, 0.06, 0.08, 0.1}.
(c) “Increased numbers of genes and causal SNPs”: This scenario involved ten risk genes (*L* = 10), each with two causal SNPs for gene expression (*M*_*el*_ = 2). In addition, we assumed ten causal SNPs not in risk genes, bringing the total number of causal SNPs to 30. The heritability was fixed at 0.2.

To make our simulations resemble real fine-mapping studies, we designed the study population to consist of 10,000 randomly selected Europeans from the UK Biobank dataset. The fine-mapping regions were randomly drawn from chromosome 1, each containing 5,000 SNPs.

Before conducting a comparative analysis of SuSiE^2^ with other methods, we evaluated the calibration accuracy of SuSiE^2^ in estimating PIPs under scenarios (*a*), (*b*), and (*c*). Scenario (*c*) allowed for a greater number of causal SNPs to be incorporated into a single simulation. In scenarios (*a*) and (*b*), the heritability was consistently set at 0.1. The simulation results, as depicted in S1 Fig, illustrate that SuSiE^2^ effectively calibrated PIPs across all scenarios. Notably, the optimal calibration was observed when the risk locus contained a substantial number of genes (scenario (*c*)).

We conducted a comprehensive comparison of five fine-mapping methods: single-trait SuSiE without eQTL information (SuSiE), SuSiE^2^ incorporating eQTL information from all risk genes (SuSiE^2^), and three multi-trait fine-mapping methods: mvSuSiE [20], flashfm [22], and fastPAINTOR [21]. For mvSuSiE and flashfm, we considered the trait-specific credible sets. However, fastPAINTOR only provides the posterior probabilities for SNPs to be casual across all traits, thus we constructed cross-trait credible sets for this method. Detailed descriptions of these methods are available in S1 Text. Throughout our simulations, we used the 95% percent CSs to capture causal variants. These methods were compared based on three key criteria:

- **Power**: The proportion of true effect SNPs included in at least one credible set.
- **Coverage**: The proportion of credible sets that contain at least one true effect variable.
- **Average size**: the average size of the credible sets detected.

**Fig 1:**
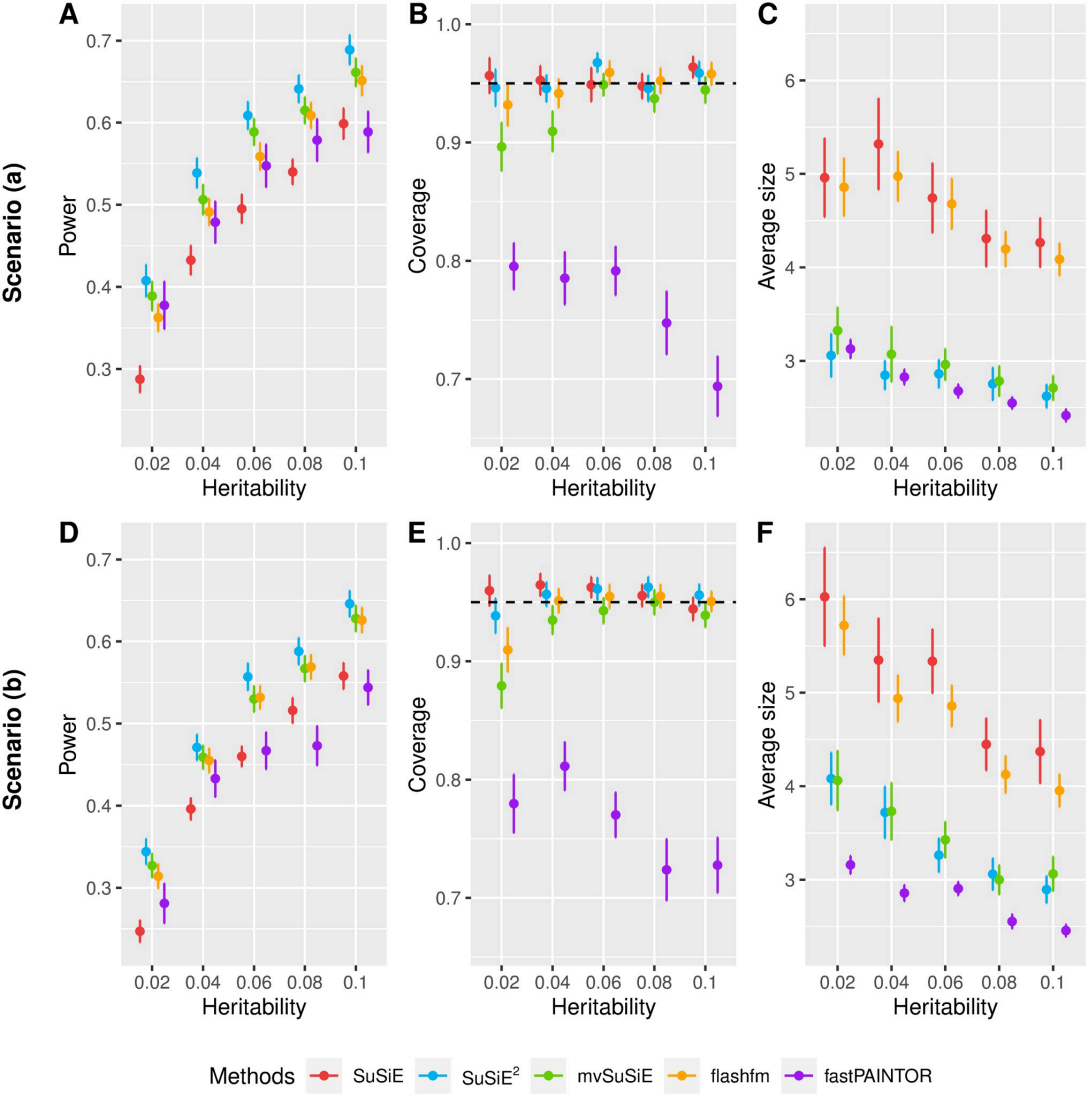
Comparison of methods in simulated data with the in-sample LD matrix. We assess the 95% credible sets generated by five fine-mapping methods (SuSiE, SuSiE^2^, mvSuSiE, flashfm, fastPAINTOR) under two scenarios (*a*) (A, B, C) and (*b*) (D, E, F). The results, averaged over 100 repetitions for each method and heritability combination, are presented with both the mean value and the empirical standard error. Panels A and D evaluate the power of detecting causal SNPs in at least one credible set. Panels B and E focus on the coverage of credible sets, with the black dashed line indicating the 95% level. Panels C and F evaluate the average size of credible sets for scenarios (*a*) and (*b*), respectively.

We first compared the fine-mapping performance utilizing summary statistics and the in-sample LD matrix, with the results summarized in Fig 1. For both scenario (*a*) and scenario (*b*), SuSiE^2^ had higher power than the other methods (Fig 1A and 1D). In comparison with single-trait SuSiE, SuSiE^2^ increased the detection rate of causal SNPs by 15% to 40%. Moreover, it had a 5% improvement compared to the second-best performing method, mvSuSiE. Notably, when the total heritability was relatively small, fastPAINTOR improved the power over single-trait SuSiE analysis. However, fastPAINTOR failed to improve the power by integrating multi-trait information over SuSiE for larger heritabilities. In both scenarios, SuSiE^2^, mvSuSiE, and flashfm enhanced the power of detecting causal SNPs over the single-trait SuSiE, but integrating gene expression level data via SuSiE^2^ yielded the most substantial improvement in power.

In all our comparisons utilizing the in-sample LD matrix, the single-trait SuSiE, SuSiE^2^, and flashfm effectively controlled the coverage at the desired 95% level (Fig 1B and 1E). However, fastPAINTOR had significantly lower coverage compared to single-trait SuSiE, SuSiE^2^, and other multi-trait fine-mapping methods (flashfm and mvSuSiE). One explanation is that fastPAINTOR only outputs the posterior probability for each SNP to be causal, thus we have to construct CSs based on those probabilities and the LD matrix. This two-step procedure led to a suboptimal coverage rate. Besides, mvSuSiE showed inadequate control of the coverage rate when the heritability was lower in both scenarios, indicating inflated false positives in the presence of weak signals.

We also evaluated the average size of CSs for all the methods (Fig 1C and 1F). As expected, single-trait SuSiE produced credible sets with the largest average size. This illustrates that incorporating gene expression levels into fine-mapping can effectively narrow down the list of potential causal SNPs. Compared with single-trait SuSiE, flashfm only reduced the average size of CSs by 5%, while SuSiE^2^ and mvSuSiE achieved reductions of 36% and 34%, respectively. It is worth mentioning that although fastPAINTOR achieved the smallest size of CSs, especially under scenario (*b*) (43% reduction compared with SuSiE), these credible sets were too narrow to reliably capture the true signal, leading to poor power and coverage.

Regardless of the selected heritability, all the single and multiple traits fine-mapping methods had better performance under scenario (*a*) compared to scenario (*b*), with higher power and smaller CS size. These findings illustrate that integration of eQTL information can be especially beneficial for fine-mapping when all the SNPs influence the phenotype by affecting gene expressions. We also investigated the robustness of SuSiE^2^ and mvSuSiE regarding the heritability for eQTL studies under scenario (*b*) in S2 Fig. Notably, we observed a substantial increase in the power and precision of SuSiE^2^ when compared to SuSiE and mvSuSiE. There was more improved power over single-trait SuSiE when the total heritability for eQTL studies was higher than 0.4% (i.e., the proportion of variation explained by each SNP is above 0.2%).

#### Fine-mapping with the 1KG dataset as reference panel

To make the simulation setting more realistic, we also evaluated the performance of the five methods when using an external reference panel from the 1000 Genomes (1KG) Phase 3 dataset [26]. We selected the European samples based on the super-population information provided by the 1KG Project, excluding all duplicated and ambiguous SNPs. We applied quality control to the 1KG data using PLINK [27], resulting in a final reference panel consisting of 503 European samples genotyped at 8,190,311 SNPs. We only considered scenario (*b*) for simulations with the 1KG reference panel.

To meet the requirement of mvSuSiE and flashfm, we first simulated phenotypes and gene expression levels from the same UKBB population. As noted by [20, 22], one limitation for mvSuSiE and flashfm is that each trait must be measured on the same population, and only one LD matrix can be used for all the traits evaluated. For a fair comparison, we utilized the 1KG dataset as the reference panel in SuSiE^2^ for both the eQTL-based SuSiE and the trait-based SuSiE. The same reference panel was also used in mvSuSiE and flashfm. However, fastPAINTOR encountered challenges in capturing any signal in most repetitions when this external reference panel was used for both phenotypes and gene expression levels. As fastPAINTOR permits multiple inputs of LD matrices for different traits, we used the in-sample reference panel for gene expression levels to improve the performance of fastPAINTOR.

**Fig 2:**
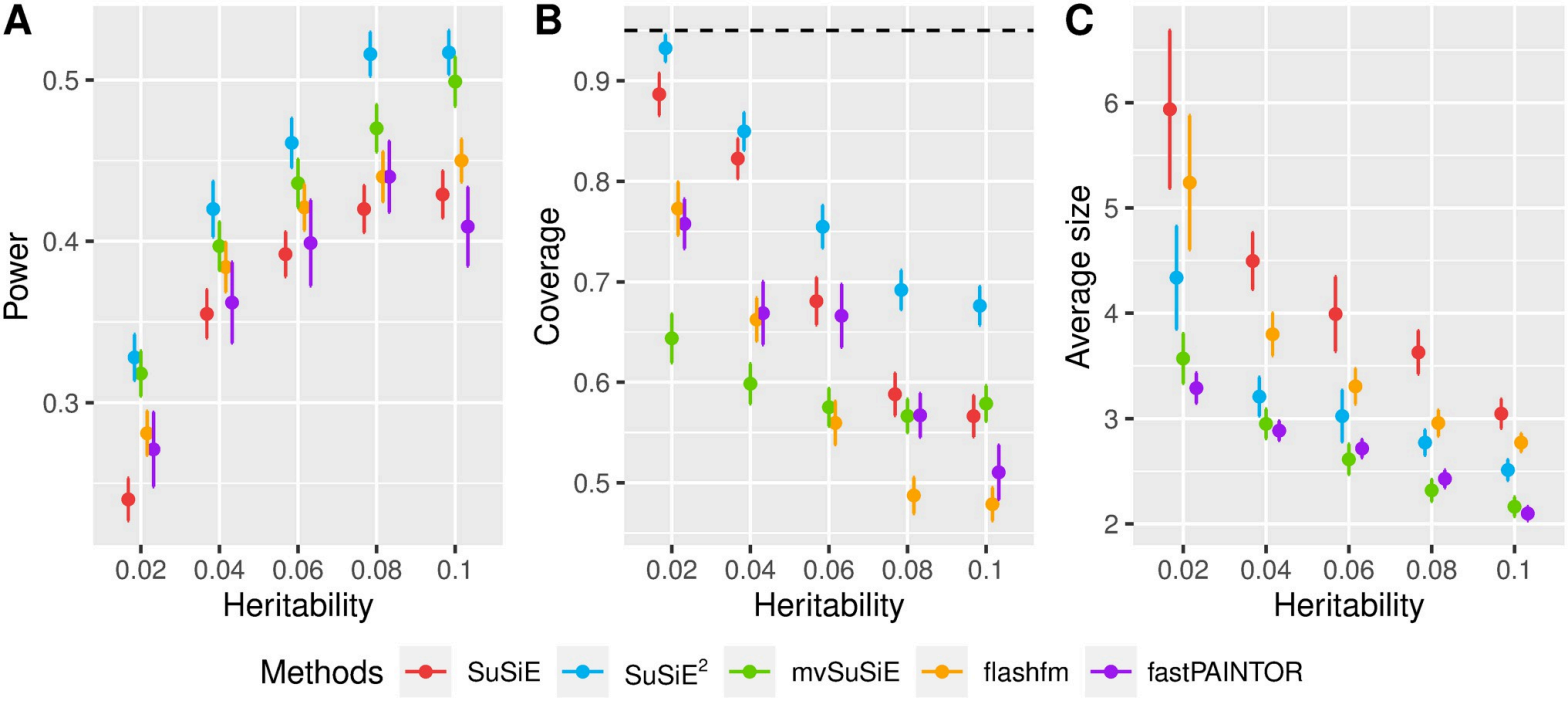
Comparison of methods in simulated data with the 1KG reference panel. We compare the 95% credible sets from five fine-mapping methods (SuSiE, SuSiE^2^, mvSuSiE, flashfm, fastPAINTOR) under scenarios (*b*). For each combination of method and heritability, we present the mean value and the standard error from 100 repetitions. Panel A gives summaries of the power of detecting causal SNPs in at least one credible set. Panel B evaluates the coverage of credible sets, with the black dashed line corresponding to the 95% level. Panel C evaluates the average size of credible sets.

We summarize the power, coverage, and average size of CSs from five methods in Fig 2. As expected, the power and coverage decreased for all fine-mapping methods when we replaced the in-sample LD matrix with the 1KG-based LD matrix. However, compared with single-trait SuSiE and other multi-trait fine-mapping methods, SuSiE^2^ still improved power for all heritability settings (Fig 2A). As shown in Fig 2B, none of the fine-mapping methods controlled the coverage of CSs at the desired level (95%), and this problem became more severe with the increase in total heritability. This phenomenon can be attributed to the tendency of fine-mapping methods to output more CSs with smaller sizes as signals become stronger (Fig 2C). With an external reference panel unable to provide precise LD patterns, it becomes more challenging for these smaller CSs to accurately capture the true signal. However, SuSiE^2^ improved coverage compared to other methods across all simulation settings, and almost reached the desired coverage of 95% when the total heritability was low (2%). In comparison, mvSuSiE had a much lower coverage than both single-trait SuSiE and SuSiE^2^, suggesting that mvSuSiE may lead to a higher proportion of false discoveries under an inconsistent LD matrix based on an external reference panel.

Different from other multi-trait fine-mapping methods, SuSiE^2^ employs a two-stage model that allows us to estimate the first-stage PIPs with the in-sample LD matrix for eQTLs, and then apply them as priors to the summary statistics of the phenotype. In the above simulation, if we substitute the 1KG-based LD matrix with the in-sample LD matrix in the eQTL-based SuSiE step, we observe a further improvement in the performance of SuSiE^2^ in terms of power and coverage (S3 Fig).

**Fig 3:**
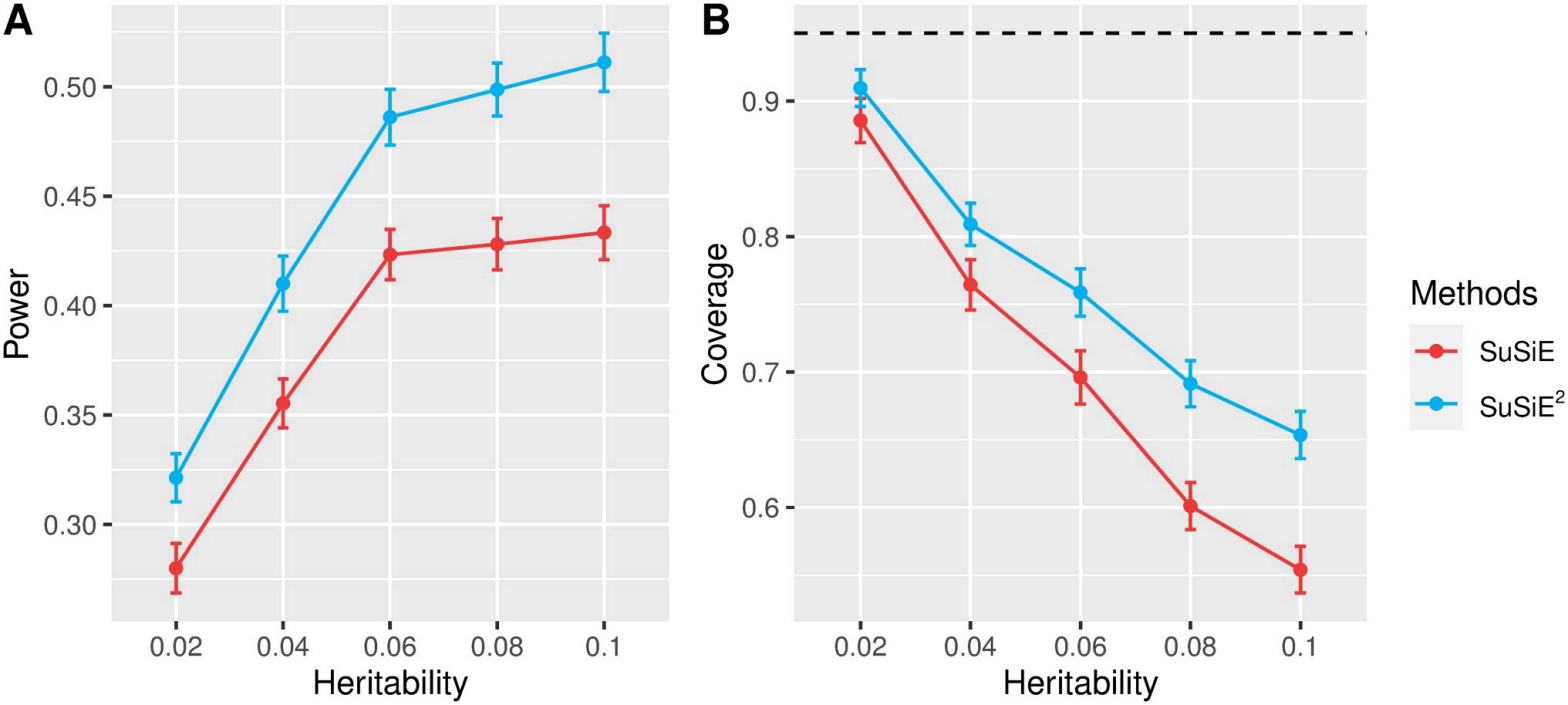
Comparison of SuSiE and SuSiE^2^ with a smaller gene expression dataset and 1KG panel. We simulated gene expression levels based on 2,000 UKBB samples, and simulated trait of interest based on 10,000 different UKBB samples under scenario (*b*). We compare the 95% credible sets from SuSiE and SuSiE^2^ from 100 repetitions. Panel A evaluates the power of detecting causal SNPs in at least one credible set. Panel B evaluates the coverage of credible sets, with the black dashed line corresponding to the 95% level.

In practical application, summary statistics for trait of interest are typically obtained from large-scale GWASs, while gene expression levels are obtained from different datasets with relatively small sample sizes. To illustrate that SuSiE^2^ can indeed improve fine-mapping performance in such scenarios, we modified scenario (*b*) by simulating gene expression levels from another 2,000 unrelated UKBB samples with European ancestry. For the first layer in SuSiE^2^ (eQTL-based SuSiE), we used the in-sample LD matrix. For the second layer (phenotype-based SuSiE), we calculated the LD matrix based on the 1KG reference panel. We compared single-trait SuSiE with SuSiE^2^ regarding to power and coverage, as summarized in Fig 3. SuSiE^2^ improved both power and coverage over single-trait SuSiE, illustrating that integrating eQTL information from limited samples can still improve fine-mapping results.

We also evaluated the performance of SuSiE^2^ when we simulated phenotypes and gene expression levels with 503 1KG samples and employed 10,000 UKBB samples as the external reference panel in S4 Fig. The total heritability was fixed at 0.1 for scenario (*b*). With a large reference panel, SuSiE and SuSiE^2^ achieved a coverage rate close to the desired level. However, all the multi-trait fine mapping methods had inflated false discoveries. In comparison to SuSiE, integrating eQTL information with SuSiE^2^ and all multi-trait fine-mapping methods increased power, with SuSiE^2^ as the second-best performing method in this evaluation. The “best” performing method under this scenario, mvSuSiE, had poorer coverage.

In summary, the simulations demonstrate the better performance of SuSiE^2^ over single-trait SuSiE and other multi-trait fine-mapping methods through integrating eQTLs to refine fine-mapping outcomes. For the in-sample LD matrix, SuSiE^2^ consistently increased the power of detecting causal SNPs while controlling coverage and improving the precision of credible sets. With an external reference panel, SuSiE^2^ improved power compared with single-trait SuSiE while effectively reducing false discoveries from multi-trait fine-mapping methods caused by inaccurate LD patterns.

### Application to BMI from UK Biobank

In this section, we applied the SuSiE^2^ pipeline to fine-map genetic variants affecting body mass index (BMI) with summary statistics from the UK Biobank GWAS imputation v3 (phenotype code: 21001). This dataset included 359,983 participants, with summary statistics at 13,791,467 SNPs. We only focused on common SNPs by filtering out SNPs with minor allele frequencies smaller than 0.01, resulting in 1,127,242 SNPs. The reference panel we used contained 20,000 unrelated UK Biobank European samples. We obtained the gene expression data from the Genotype-Tissue Expression (GTEx v8) Project [28], which provided the tissue-specific gene expression levels and whole-genome sequencing data. To aid in the fine-mapping of BMI, we integrated eQTL information from two tissues: subcutaneous adipose (ADS, UBERON:0002190) and visceral adipose (ADV, UBERON:0010414). The number of samples with genotype data for ADS and ADV were 581 and 469, respectively.

Based on the BMI summary statistics, we obtained a total of 714 candidate regions for fine-mapping. These candidate regions were constructed around significant genetic association signals (*p*-value < 5 × 10^−7^), with the property that all SNPs within 50 Kb upstream and downstream of each signal belonged to the same candidate region. We also combined overlapping regions until there was no more overlap. We then applied the SuSiE^2^ pipeline to these 714 candidate regions for BMI and compared the results with SuSiE, excluding the eQTL information. For both SuSiE and SuSiE^2^, the number of signals in each risk region was assumed to be 5.

**Fig 4:**
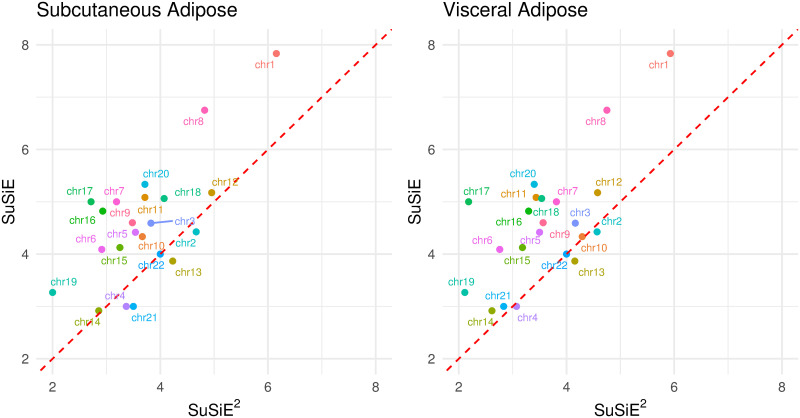
Average size of credible sets by SuSiE and SuSiE^2^. We applied the SuSiE^2^ pipeline with gene expression from subcutaneous adipose (left) and visceral adipose (right). The 95% credible sets were grouped according to the chromosome in which they are located, labeled by chr*i* for the *i*th chromosome. The minimum absolute correlation allowed in a credible set was fixed at 0.9 for both SuSiE and SuSiE^2^.

From the 714 candidate regions, SuSiE identified 449 95% credible sets. The average size of these credible sets was 4.8. Among these credible sets, 138 comprised only one SNP (“1-SNP CS”), and 290 contained fewer than five SNPs (“<5-SNP CS”). When incorporating gene expression data from ADS into SuSiE^2^, the number of identified credible sets increased to 480. The counts of “1-SNP CS” and “<5-SNP CS” also increased to 165 and 340, respectively. Integration of gene expression data from ADV yielded similar results, as summarized in Table 1. Compared with SuSiE, SuSiE^2^ reduced the average size of credible sets to 3.86 and 3.82 by integrating gene expression data from ADS and ADV, respectively. Additionally, the median size of credible sets was also reduced from 3 SNPs to 2 SNPs by SuSiE^2^ using eQTL information from either of these two tissues. Fig 4 provides a comparison of the average size of credible sets obtained from SuSiE and SuSiE^2^ grouped by chromosome. For most chromosomes, the average size of credible sets from SuSiE^2^ was smaller, illustrating that incorporating eQTL information via SuSiE^2^ can improve fine-mapping precision. Besides, the results with ADS and ADV exhibit a similar pattern, suggesting that eQTL information from different adipose tissues consistently contributes to improving fine-mapping results.

**Table 1:** Summary of credible sets detected by SuSiE and SuSiE^2^ in the BMI study.

Method	Total	1-SNP CS	<5-SNP CS	Average size	Median size
SuSiE	449	138	290	4.80	3
SuSiE^2^ (ADS)	480	165	340	3.86	2
SuSiE^2^ (ADV)	461	162	338	3.82	2

**Fig 5:**
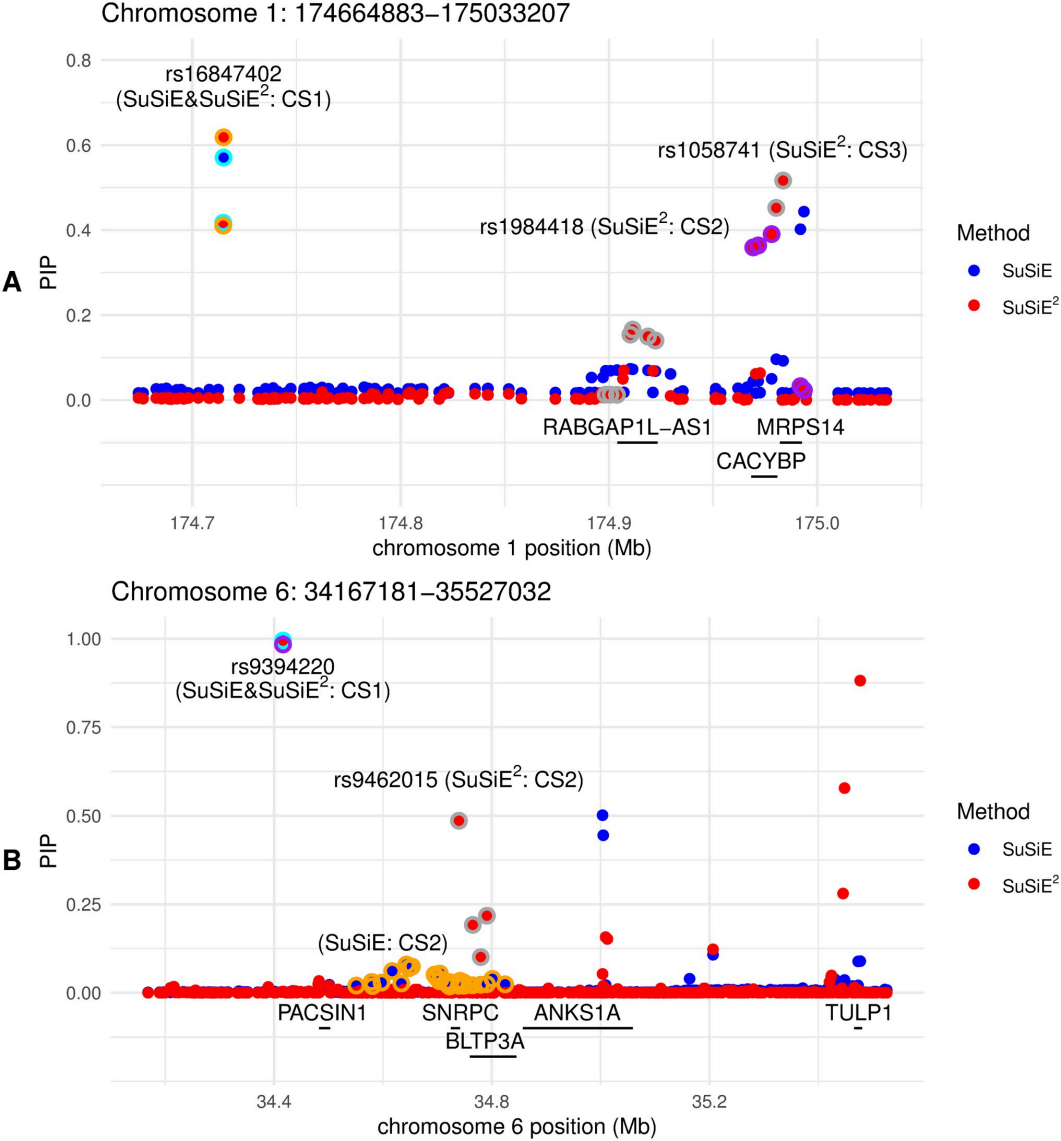
Fine-mapping examples on BMI risk loci with SuSiE and SuSiE^2^. The plots show the estimated PIPs for each SNP in two risk regions by SuSiE and SuSiE^2^. Panel A presents the results for a risk region on chromosome 1. Panel B illustrates the results for a risk region on chromosome 6. The SNPs from the same 95% credible sets by SuSiE or SuSiE^2^ are surrounded by circles in the same corresponding color. We label each credible set with the SNP ID of the leading variant.

To illustrate the potential for SuSiE^2^ to help detect signals for complex traits and reduce the size of credible sets, we investigated two example risk regions in more detail, as shown in Fig 5. The first region involves multiple genes, including the MRPS14 and CACYBP (Fig 5A). SNPs in MRPS14 and CACYBP have previously been associated with BMI [29–32]. Within this region, SuSiE identified only one credible set containing two SNPs (CS1), which was also identified by SuSiE^2^. After integrating gene expression data from the ADS tissue, SuSiE^2^ identified two additional signals: one with the leading SNP rs1984418 in CACYBP (CS2; size: 5; purity: 0.983), and another with the leading SNP rs1058741 in MRPS14 (CS3; size: 9; purity: 0.966). This example illustrates that integrating eQTL information via SuSiE^2^ has the potential for more discoveries by prioritizing functional variants within gene regions. The second region contains multiple genes around SNRPC (Fig 5B), a gene locus with several well-studied variants associated with BMI and obesity [31, 33]. Both SuSiE and SuSiE^2^ identified the same 1-SNP credible set (CS1) containing rs9394220, located around PACSIN1. However, for the second signal around the SNRPC locus, SuSiE selected a credible set containing 28 SNPs (purity: 0.989), with no significant leading variant. In contrast, integrating gene expression levels from the ADS tissue reduced this credible set to only 4 SNPs (purity: 0.999), with the leading variant rs9462015. This result suggests that SuSiE^2^ can improve fine-mapping precision by selecting the most likely risk variants from a large credible set based on the probability of being an eQTL.

### Application to AD dataset

In this section, we applied SuSiE^2^ to a real dataset on Alzheimer’s disease. The summary statistics we used were from a recent meta-analysis of individuals from 13 cohorts, with a total of 1,126,563 individuals (90,338 cases and 1,036,225 controls) included [34]. This meta-analysis identified 3,915 significant (*P* < 10^−8^) variants across 38 independent loci, including seven loci that had not been reported previously. The sample size generating the summary statistic for each SNP ranged from 216 to 762,917, with a median of 661,401. To make the z-scores of each SNP more comparable, we removed those SNPs with corresponding sample sizes smaller than 500,000, leaving 7,578,057 out of 12,688,308 variants.

We obtained the gene expression levels for AD risk loci from the ROSMAP dataset [35], which contained the bulk RNA sequencing (RNA-seq) data of 642 individuals. Among them, 473 individuals also had genotype data available on 572,266 SNPs, which allowed us to conduct an eQTL study for AD risk loci via SuSiE. We used the Michigan imputation server [36] with 1000 Genomes Phase 3 (Version 5) as the reference panel. After imputation, we obtained the genotype data for 473 ROSMAP samples at 13,753,668 SNPs.

To evaluate our method, we treated the predicted functional SNPs for Alzheimer’s disease from a single-cell epigenomic analysis [37] as the validation data. This study developed a machine-learning classifier to integrate a multi-omic framework and identified multiple pairs of AD risk locus and the most likely mediator in both coding and non-coding regions. After removing the APOE locus because of multiple mediators, there were in total 35 pairs of AD risk locus and mediator, 16 in the coding regions and 19 in the non-coding regions.

Our real data analysis was conducted with the following steps:

1. We extracted all the common SNPs within 100kb upstream and downstream of each likely mediator as a target set.
2. The LD matrix was calculated for each target set with a reference panel based on Europeans from the UKBB dataset.
3. We fitted the eQTL-based SuSiE model with the ROSMAP dataset and calculated the PIP for each candidate SNP in the target set.
4. PIPs from step 3 were treated as prior distributions and integrated into the fine-mapping study based on summary statistics from the meta-analysis to get SuSiE^2^ results.

Two fine-mapping methods we considered were SuSiE^2^ and the original SuSiE that did not take advantage of the eQTL information. We only considered 20 mediator-risk loci pairs in the common part of the ROSMAP dataset, reference panel, and the meta-analysis dataset. We compared the AD mediators identified by SuSiE and SuSiE^2^ (captured in at least one credible set by SuSiE and SuSiE^2^), with the results summarized in Table 2. SuSiE^2^ successfully identified nine out of 20 mediators, while SuSiE only captured five of them. In the coding region, there were in total seven causal SNPs, SuSiE identified two of them, while SuSiE^2^ detected three of them. In the non-coding region, the number of AD mediators identified by SuSiE was three, while the number of mediators identified by SuSiE^2^ was six. We also evaluated the properties of generated credible sets (CSs) by two methods, summarized in Table 3. The original SuSiE captured 27 credible sets, with an average size of 9.6, while integrating eQTL information allowed us to identify 29 credible sets and reduced the average size to 8.0. Compared with SuSiE, SuSiE^2^ also reduced the 75% quantile of the size of credible sets from 13 to 11, which suggests that SuSiE^2^ may avoid producing extremely large credible sets.

**Table 2:** Summary of AD mediators detected by SuSiE and SuSiE^2^.

Method	Total	Identified (Total)	Coding Region	Identified (Coding region)
SuSiE	20	5	7	2
SuSiE^2^	20	9	7	3

**Table 3:** Summary of credible sets identified by SuSiE and SuSiE^2^.

Method	Number of CS	Average Size	25% Quantile	Median	75% Quantile
SuSiE	27	9.6	2	4	13
SuSiE^2^	29	8.0	2	4	11

**Fig 6:**
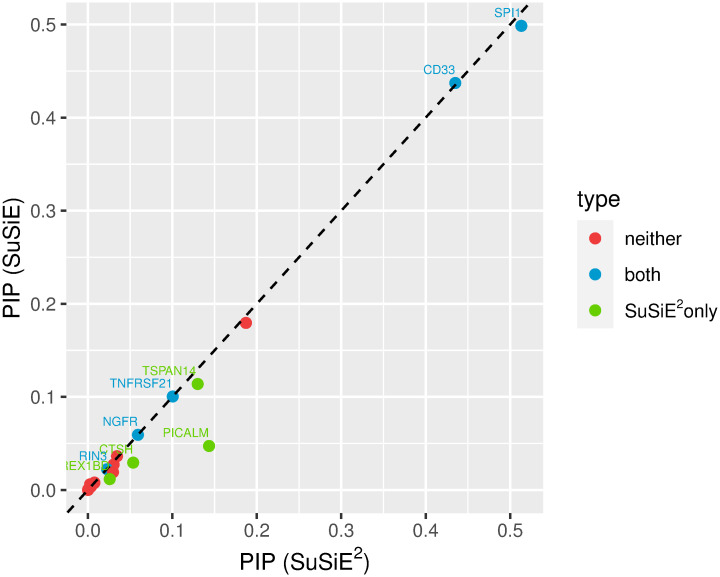
Estimated PIP for each AD mediator by SuSiE and SuSiE^2^. There were in total 20 AD risk loci divided into the following three categories. Five mediators were captured by both SuSiE and SuSiE^2^ in one credible set, denoted by the blue dots. SuSiE^2^ identified four additional risk loci, denoted by the green dots. The remaining 11 loci could not be captured in any credible set by either SuSiE or SuSiE^2^, corresponding to the red dots.

We also calculated the PIP for each mediator by SuSiE and SuSiE^2^, as shown in Fig 6. From this plot, we observed that SuSiE^2^ can identify more AD mediators by increasing the estimated PIPs of them, and all the mediators identified by SuSiE were also captured by SuSiE^2^. Besides, the points of many causal SNPs were distributed around the *y* = *x* line, which suggests that the SuSiE regression model may not be very sensitive to the choice of prior probabilities. The numerical results of PIPs estimated by SuSiE and SuSiE^2^ for every AD mediator are summarized in S1 Table. Based on both Fig 6 and S1 Table, SuSiE^2^ improved the estimated PIPs of the most-likely mediator for some AD risk genes, such as PICALM, CTSH, and REX1BD. However, for other mediators, the PIPs estimated by SuSiE^2^ were not significantly different from those estimated by SuSiE. Our explanation is that for some AD risk genes, we cannot obtain informative priors from the eQTL-based SuSiE model, either because of weak eQTL signals or the small sample size. In some extreme cases, such as TNFRSF21, TMEM139, and NGFR, the PIPs estimated by the eQTL-based SuSiE were equal for all candidate SNPs. For these AD genes, the results of SuSiE^2^ were exactly the same as the results of SuSiE.

**Fig 7:**
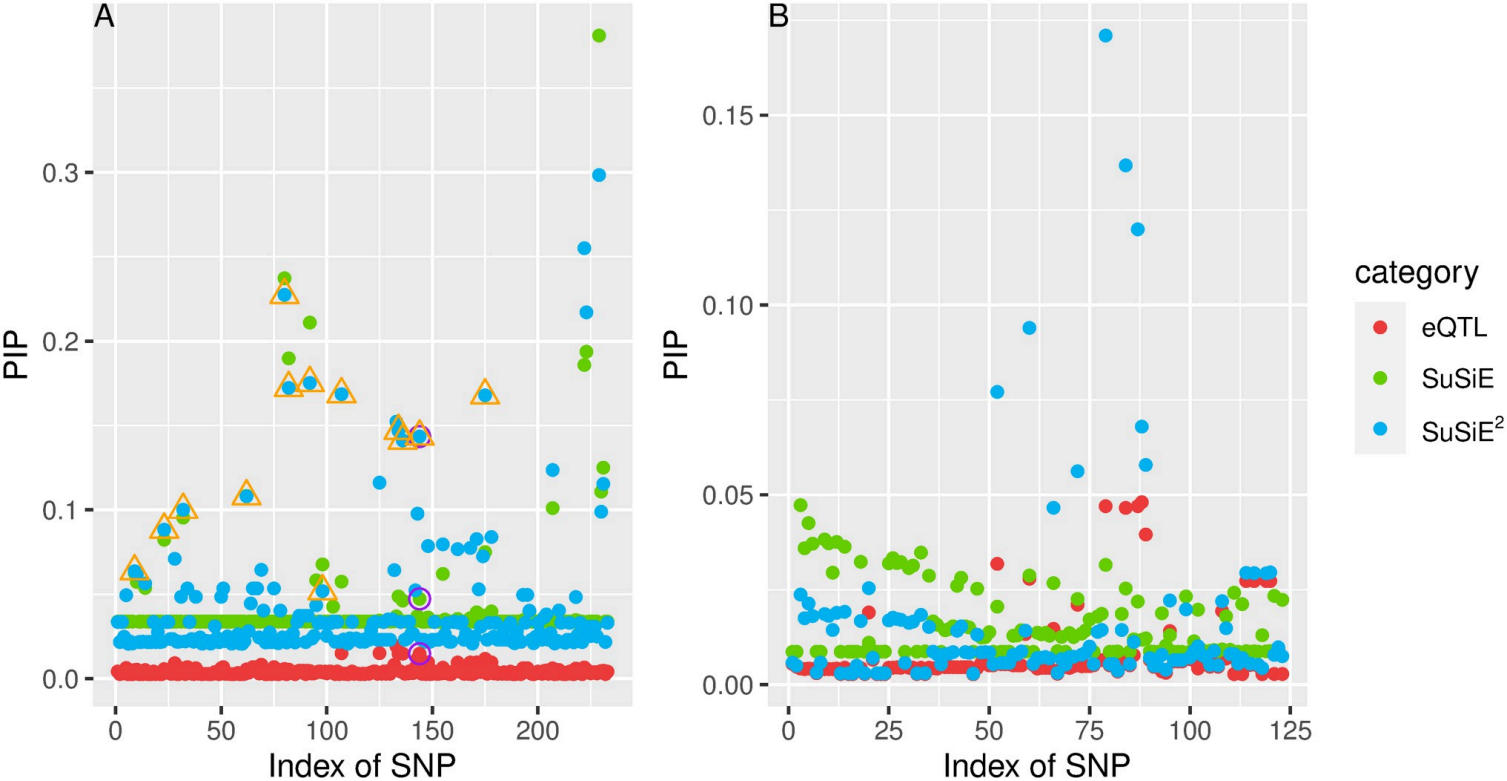
Estimated PIPs by SuSiE, SuSiE^2^ and eQTL-based SuSiE for PICALM (A) and C14orf93 (B). The PIPs estimated from the eQTL study are used as the prior information by SuSiE for SuSiE^2^. For the PICALM locus, PIPs for the true mediator in this locus are surrounded by the purple circle, and the points surrounded by an orange triangle correspond to the credible set from SuSiE^2^ which can capture the true mediator. For the C14orf93 locus, the true mediator was not included in the common part of summary statistics and ROSMAP data.

To illustrate that SuSiE^2^ enhanced the PIPs for some of the causal mediators, we display the examples of two risk loci in Fig 7. We considered the PIPs for all variants within these loci from the following three categories: eQTL study, SuSiE, and SuSiE^2^. The PIPs estimated from the eQTL study are used as the prior information in SuSiE^2^. For the PICALM locus (Fig 7A), a slightly larger PIP was assigned to the true AD mediator compared with most candidate variants by the eQTL-based SuSiE, which allowed SuSiE^2^ to capture this mediator in a credible set. However, the original SuSiE failed to include this variant in any credible sets. For the C14orf93 locus (Fig 7B), both SuSiE and SuSiE^2^ failed to find any signal in the risk locus. The estimated PIPs by SuSiE were stable at a very low level, with the largest PIP smaller than 0.05. In contrast, with the prior information provided by the eQTL study, the signals for some candidate SNPs in this region were enhanced, with the strongest PIP larger than 0.15. Besides, the PIPs for the remaining SNPs estimated by SuSiE^2^ were reduced towards zero, which indicated that SuSiE^2^ performed better in separating causal SNPs from non-causal variants.

In conclusion, the real data analysis results on BMI and AD suggest that incorporating eQTL information via the SuSiE^2^ pipeline can lead to more discoveries by prioritizing functional variants within gene regions and increase the precision of fine-mapping by reducing the average size of credible sets. Besides, SuSiE^2^ achieved a better performance in separating causal SNPs from non-causal SNPs.

## Discussion

Statistical fine-mapping has been an important tool in detecting the true causal SNPs for complex traits of interest. Most widely used fine-mapping methods are based on the Bayesian framework, where assigning a proper prior distribution to risk variants can enhance both accuracy and efficiency. An effective strategy to prioritize functional variants within the risk region is to assess their associations with gene expressions. The integration of eQTL information is expected to further improve the fine-mapping performance. In this manuscript, we proposed a novel framework for integrating eQTL with fine-mapping via the SuSiE model. Through the simulation study, we demonstrated that SuSiE^2^ can increase the statistical power and precision of fine-mapping while controlling the coverage of credible sets. The advantage of SuSiE^2^ over single-trait SuSiE and multi-trait fine-mapping methods was demonstrated with comprehensive simulations using either the in-sample LD matrix or an external reference panel. The real data applications to BMI and AD showed that SuSiE^2^ outperformed single-trait SuSiE in identifying causal signals while reducing the size of credible sets by prioritizing risk variants based on eQTL information before conducting the fine-mapping study.

In the original SuSiE, the authors proved the co-ordinate ascent algorithm in the IBSS method converges to a stationary point provided that $0<\sigma^{2},\sigma_{0}^{2}<\infty$, *π*_*j*_ > 0 for all *j* = 1, …, *p* [8]. Since SuSiE^2^ is a two-stage extension of SuSiE, it shares the same theoretical convergency property. As a noteworthy observation, it happens when the PIPs estimated by the eQTL-based SuSiE equal to zero for some SNPs. However, our empirical findings indicate that the presence of these zero priors does not lead to convergence issues for the subsequent phenotype-based SuSiE step. In the rare case of encountering convergence problems, we can replace these zero priors with a small positive value without altering the final results. For instance, in the BMI analysis, modifying the zero priors with a positive value of 1 × 10^−10^ did not change the outcomes compared to the unmodified approach.

Several challenges remain to be addressed in the future. The first one is that SuSiE^2^ asks the user to decide which eQTL information to include, which may require additional variable selections. One possibility is to integrate expression levels of all genes within the risk region from relevant tissues or cell types. Second, although our simulation suggests that SuSiE is generally robust to overstating of the total number of causal effects *K* in the IBSS algorithm [8], SuSiE is not very stable to the choice of *K* in real data applications. A larger *K* sometimes leads to the finding of new credible sets. Based on our experience, we recommend increasing the parameter *K* starting from 1 and stopping this process when we fail to find new credible sets. Further investigation of the mechanisms underlying this phenomenon is needed to find the best way to select the parameter and make use of the prior information. With the SuSiE^2^ framework, there exists the potential to jointly consider eQTLs across multiple tissues and incorporate additional molecular QTL information to more comprehensively capture different mechanisms contributing to diseases.

## Conclusion

In this manuscript, we have introduced SuSiE^2^, a two-layer statistical framework that incorporates eQTL information into fine-mapping. By prioritizing variants within the candidate region with eQTL information, SuSiE^2^ improved the performance of fine-mapping by increasing statistical power while reducing the average size of credible sets compared to the single-trait SuSiE. Through simulations with an external reference panel, we also demonstrated that eQTL information can compensate for the coverage loss caused by inaccurate LD information compared with other multi-trait fine-mapping methods. In real data applications, SuSiE^2^ identified more causal signals for BMI and implicated four additional SNPs associated with AD in comparison to SuSiE. Evaluations of fine-mapping examples for BMI and AD suggest that SuSiE^2^ enhances fine-mapping performance by prioritizing functional variants within gene regions.

## Supporting information

S1 Text Supplementary notes. (PDF)

S1 Table Summary information of AD mediators. (PDF)

S1 Fig Assessment of PIP calibration by SuSiE^2^. In each scenario, we repeated the simulation for 1,000 times and grouped the SNPs into 10 evenly spaced bins from 0 to 1 according to their PIP estimated by SuSiE^2^. The x-axis of this calibration figure is the average of predicted PIP for each bin, and the y-axis is the fraction of causal SNPs in each bin. The dashed line corresponds to the *y* = *x* diagonal line. A well-calibrated method should produce points close to the diagonal line.
(TIF)

S2 FigRobustness of SuSiE^2^ and mvSuSiE to the heritability for eQTL study.We compare the 95% credible sets from SuSiE, SuSiE^2^, and mvSuSiE under scenario (*b*). The heritability of direct effect for phenotype ($1-\sigma_{t}^{2}$) is fixed at 0.1. The heritability for the eQTL study ($1-\sigma_{el}^{2}$) increased from 0.001 to 0.02. Panel A evaluates the power of detecting causal SNPs in at least one credible set. Panel B evaluates the coverage of credible sets, with the black dashed line corresponding to the 95% level. Panel C evaluates the average size of credible sets.(TIF)

S3 FigComparison of SuSiE^2^ when using in-sample or external LD matrices for the first step of SuSiE^2^.We compare the 95% credible sets from SuSiE^2^ when using either the in-sample LD matrix (${\text{SuSiE}}_{i}^{2}$) or the LD matrix from 1KG panel (${\text{SuSiE}}_{e}^{2}$) in the eQTL-based SuSiE. For each combination of method and heritability, we show the mean value and the standard error from 150 repetitions. Panel A evaluates the power of detecting causal SNPs in at least one credible set. Panel B evaluates the coverage of credible sets, with the black dashed line corresponding to the 95% level. For the second step of SuSiE^2^ (fine-mapping for the trait of interest), we always used the LD matrix from the 1KG reference panel.(TIF)

S4 Fig Comparison of fine-mapping results when using 1KG simulated data and the UKBB reference panel. We compare the 95% credible sets from five fine-mapping methods (SuSiE, SuSiE^2^, mvSuSiE, flashfm, fastPAINTOR) under scenario (*b*). The total heritability was fixed at 0.1. For each combination of method and heritability, we show the mean value and the empirical standard error from 150 repetitions. Panel A evaluates the power of detecting causal SNPs in at least one credible set. Panel B evaluates the coverage of credible sets, with the black dashed line corresponding to the 95% level. Phenotype and gene expression levels were simulated based on the genotypes of 5,000 SNPs from 503 1KG samples. The reference panel consisted of 10,000 UKBB samples.
(TIF)

## References

[1] Visscher PM, Brown MA, McCarthy MI, Yang J. Five years of GWAS discovery. The American Journal of Human Genetics. 2012;90(1):7–24. doi: 10.1016/j.ajhg.2011.11.029 22243964 PMC3257326

[2] Schaid DJ, Chen W, Larson NB. From genome-wide associations to candidate causal variants by statistical fine-mapping. Nature Reviews Genetics. 2018;19(8):491–504. doi: 10.1038/s41576-018-0016-z 29844615 PMC6050137

[3] Spain SL, Barrett JC. Strategies for fine-mapping complex traits. Human molecular genetics. 2015;24(R1):R111–R119. doi: 10.1093/hmg/ddv260 26157023 PMC4572002

[4] Maller JB, McVean G, Byrnes J, Vukcevic D, Palin K, Su Z, et al. Bayesian refinement of association signals for 14 loci in 3 common diseases. Nature genetics. 2012;44(12):1294–1301. doi: 10.1038/ng.2435 23104008 PMC3791416

[5] (IIBDGC) IIGC, Agliardi C, Alfredsson L, Alizadeh M, Anderson C, Andrews R, et al. Analysis of immune-related loci identifies 48 new susceptibility variants for multiple sclerosis. Nature genetics. 2013;45(11):1353–1360. doi: 10.1038/ng.2770 24076602 PMC3832895

[6] Hormozdiari F, Kostem E, Kang EY, Pasaniuc B, Eskin E. Identifying causal variants at loci with multiple signals of association. In: Proceedings of the 5th ACM Conference on Bioinformatics, Computational Biology, and Health Informatics; 2014. p. 610–611. 10.1534/genetics.114.167908 PMC4196608 25104515

[7] Benner C, Spencer CC, Havulinna AS, Salomaa V, Ripatti S, Pirinen M. FINEMAP: efficient variable selection using summary data from genome-wide association studies. Bioinformatics. 2016;32(10):1493–1501. doi: 10.1093/bioinformatics/btw018 26773131 PMC4866522

[8] Wang G, Sarkar A, Carbonetto P, Stephens M. A simple new approach to variable selection in regression, with application to genetic fine mapping. Journal of the Royal Statistical Society Series B: Statistical Methodology. 2020;82(5):1273–1300. doi: 10.1111/rssb.12388 37220626 PMC10201948

[9] Mitchell TJ, Beauchamp JJ. Bayesian variable selection in linear regression. Journal of the american statistical association. 1988;83(404):1023–1032. doi: 10.1080/01621459.1988.10478694

[10] GTEx Consortium, Ardlie KG, Deluca DS, Segrè AV, Sullivan TJ, Young TR, et al. The Genotype-Tissue Expression (GTEx) pilot analysis: multitissue gene regulation in humans. Science. 2015;348(6235):648–660. doi: 10.1126/science.1262110 25954001 PMC4547484

[11] Wen X, Lee Y, Luca F, Pique-Regi R. Efficient integrative multi-SNP association analysis via deterministic approximation of posteriors. The American Journal of Human Genetics. 2016;98(6):1114–1129. doi: 10.1016/j.ajhg.2016.03.029 27236919 PMC4908152

[12] Nicolae DL, Gamazon E, Zhang W, Duan S, Dolan ME, Cox NJ. Trait-associated SNPs are more likely to be eQTLs: annotation to enhance discovery from GWAS. PLoS genetics. 2010;6(4):e1000888. doi: 10.1371/journal.pgen.1000888 20369019 PMC2848547

[13] Giambartolomei C, Vukcevic D, Schadt EE, Franke L, Hingorani AD, Wallace C, et al. Bayesian test for colocalisation between pairs of genetic association studies using summary statistics. PLoS genetics. 2014;10(5):e1004383. doi: 10.1371/journal.pgen.1004383 24830394 PMC4022491

[14] Hormozdiari F, Van De Bunt M, Segre AV, Li X, Joo JWJ, Bilow M, et al. Colocalization of GWAS and eQTL signals detects target genes. The American Journal of Human Genetics. 2016;99(6):1245–1260. doi: 10.1016/j.ajhg.2016.10.003 27866706 PMC5142122

[15] Wallace C. A more accurate method for colocalisation analysis allowing for multiple causal variants. PLoS genetics. 2021;17(9):e1009440. doi: 10.1371/journal.pgen.1009440 34587156 PMC8504726

[16] Kichaev G, Yang WY, Lindstrom S, Hormozdiari F, Eskin E, Price AL, et al. Integrating functional data to prioritize causal variants in statistical fine-mapping studies. PLoS genetics. 2014;10(10):e1004722. doi: 10.1371/journal.pgen.1004722 25357204 PMC4214605

[17] Weissbrod O, Hormozdiari F, Benner C, Cui R, Ulirsch J, Gazal S, et al. Functionally informed fine-mapping and polygenic localization of complex trait heritability. Nature genetics. 2020;52(12):1355–1363. doi: 10.1038/s41588-020-00735-5 33199916 PMC7710571

[18] Zhang W, Najafabadi H, Li Y. SparsePro: an efficient genome-wide fine-mapping method integrating summary statistics and functional annotations. bioRxiv. 2021; p. 2021–10. 10.1371/journal.pgen.1011104 PMC10781022 38153934

[19] Yang Z, Wang C, Liu L, Khan A, Lee A, Vardarajan B, et al. CARMA is a new Bayesian model for fine-mapping in genome-wide association meta-analyses. Nature Genetics. 2023; p. 1–9. 37169873 10.1038/s41588-023-01392-0

[20] Zou Y, Carbonetto P, Xie D, Wang G, Stephens M. Fast and flexible joint fine-mapping of multiple traits via the Sum of Single Effects model. bioRxiv. 2023; p. 2023–04. doi: 10.1101/2023.04.14.536893 37425935 PMC10327118

[21] Hernández N, Soenksen J, Newcombe P, Sandhu M, Barroso I, Wallace C, et al. The flashfm approach for fine-mapping multiple quantitative traits. Nature Communications. 2021;12(1):6147. doi: 10.1038/s41467-021-26364-y 34686674 PMC8536717

[22] Kichaev G, Roytman M, Johnson R, Eskin E, Lindstroem S, Kraft P, et al. Improved methods for multi-trait fine mapping of pleiotropic risk loci. Bioinformatics. 2017;33(2):248–255. doi: 10.1093/bioinformatics/btw615 27663501 PMC5254076

[23] Bottolo L, Petretto E, Blankenberg S, Cambien F, Cook SA, Tiret L, et al. Bayesian detection of expression quantitative trait loci hot spots. Genetics. 2011;189(4):1449–1459. doi: 10.1534/genetics.111.131425 21926303 PMC3241411

[24] Servin B, Stephens M. Imputation-based analysis of association studies: candidate regions and quantitative traits. PLoS genetics. 2007;3(7):e114. doi: 10.1371/journal.pgen.0030114 17676998 PMC1934390

[25] Zou Y, Carbonetto P, Wang G, Stephens M. Fine-mapping from summary data with the “Sum of Single Effects” model. PLoS Genetics. 2022;18(7):e1010299. doi: 10.1371/journal.pgen.1010299 35853082 PMC9337707

[26] Consortium GP, Auton A, Brooks L, Durbin R, Garrison E, Kang H. A global reference for human genetic variation. Nature. 2015;526(7571):68–74. doi: 10.1038/nature15393 26432245 PMC4750478

[27] Purcell S, Neale B, Todd-Brown K, Thomas L, Ferreira MA, Bender D, et al. PLINK: a tool set for whole-genome association and population-based linkage analyses. The American journal of human genetics. 2007;81(3):559–575. doi: 10.1086/519795 17701901 PMC1950838

[28] GTEx Consortium. The GTEx Consortium atlas of genetic regulatory effects across human tissues. Science. 2020;369(6509):1318–1330. doi: 10.1126/science.aaz1776 32913098 PMC7737656

[29] Huang J, Huffman JE, Huang Y, Do Valle Í, Assimes TL, Raghavan S, et al. Genomics and phenomics of body mass index reveals a complex disease network. Nature Communications. 2022;13(1):7973. doi: 10.1038/s41467-022-35553-2 36581621 PMC9798356

[30] Kichaev G, Bhatia G, Loh PR, Gazal S, Burch K, Freund MK, et al. Leveraging polygenic functional enrichment to improve GWAS power. The American Journal of Human Genetics. 2019;104(1):65–75. doi: 10.1016/j.ajhg.2018.11.008 30595370 PMC6323418

[31] Pulit SL, Stoneman C, Morris AP, Wood AR, Glastonbury CA, Tyrrell J, et al. Meta-analysis of genome-wide association studies for body fat distribution in 694 649 individuals of European ancestry. Human molecular genetics. 2019;28(1):166–174. doi: 10.1093/hmg/ddy327 30239722 PMC6298238

[32] Zhu Z, Guo Y, Shi H, Liu CL, Panganiban RA, Chung W, et al. Shared genetic and experimental links between obesity-related traits and asthma subtypes in UK Biobank. Journal of Allergy and Clinical Immunology. 2020;145(2):537–549. doi: 10.1016/j.jaci.2019.09.035 31669095 PMC7010560

[33] Locke AE, Kahali B, Berndt SI, Justice AE, Pers TH, Day FR, et al. Genetic studies of body mass index yield new insights for obesity biology. Nature. 2015;518(7538):197–206. doi: 10.1038/nature14177 25673413 PMC4382211

[34] Wightman DP, Jansen IE, Savage JE, Shadrin AA, Bahrami S, Holland D, et al. A genome-wide association study with 1,126,563 individuals identifies new risk loci for Alzheimer’s disease. Nature genetics. 2021;53(9):1276–1282. doi: 10.1038/s41588-021-00921-z 34493870 PMC10243600

[35] Bennett DA, Buchman AS, Boyle PA, Barnes LL, Wilson RS, Schneider JA. Religious orders study and rush memory and aging project. Journal of Alzheimer’s disease. 2018;64(s1):S161–S189. doi: 10.3233/JAD-179939 29865057 PMC6380522

[36] Das S, Forer L, Schönherr S, Sidore C, Locke AE, Kwong A, et al. Next-generation genotype imputation service and methods. Nature genetics. 2016;48(10):1284–1287. doi: 10.1038/ng.3656 27571263 PMC5157836

[37] Corces MR, Shcherbina A, Kundu S, Gloudemans MJ, Frésard L, Granja JM, et al. Single-cell epigenomic analyses implicate candidate causal variants at inherited risk loci for Alzheimer’s and Parkinson’s diseases. Nature genetics. 2020;52(11):1158–1168. doi: 10.1038/s41588-020-00721-x 33106633 PMC7606627

