# Supplementary material for: Integration of expression QTLs with fine mapping via SuSiE
Source: PLoS Genet. 2024 Jan 25;20(1):e1010929. doi: 10.1371/journal.pgen.1010929 (PMC10846745; doi:10.1371/journal.pgen.1010929)
Supplement: S1 Text — (PDF) [file pgen.1010929.s001.pdf]

Supplementary Notes for "Integration of Expression QTLs with fine mapping via SuSiE"

Xiangyu Zhang[1], Wei Jiang[1], and Hongyu Zhao[1*]

1 Department of Biostatistics, School of Public Health, Yale University, New Haven, Connecticut, United States of America

* hongyu.zhao@yale.edu

1 Theoretical justification for SuSiE^2.

We first state two properties given by the SuSiE manuscript [1]:

Property 1: Bayes Factor (BF) for simple linear regression (SuSiE Appendix A.1.). Suppose a Bayesian simple linear regression model:

$$\mathbf{y} = \mathbf{x}b + \mathbf{e}, \quad \mathbf{e} \sim N(0, \sigma^2 I_n), \quad b \sim N(0, \sigma_0^2), \tag{S.1}$$

where $\mathbf{y}$ is an n-vector of response data, $\mathbf{x}$ is an n-vector of an explanatory variable, both $\mathbf{x}$ and $\mathbf{y}$ are centered to have zero mean.

Define $\hat{b} := (\mathbf{x^T x})^{-1}\mathbf{x^T y}$ is the least-squared estimate of b, $s^2 := \sigma^2/(\mathbf{x^T x})$ is the variance of $\hat{b}$, and $z := \hat{b}/s$ is the corresponding z score. Then the Bayes Factor (BF) for comparing this model with the null model ($b = 0$) is:

$$BF(\mathbf{x}, \mathbf{y}; \sigma^2, \sigma_0^2) := \frac{p(\mathbf{y}|\mathbf{x}, \sigma^2, \sigma_0^2)}{p(\mathbf{y}|\mathbf{x}, \sigma^2, b = 0)} = \sqrt{\frac{s^2}{s^2 + \sigma_0^2}} exp\left(\frac{z^2}{2} \cdot \frac{\sigma_0^2}{s^2 + \sigma_0^2}\right). \tag{S.2}$$

Property 2: The single effect regression (SER) model (SuSiE Appendix A.2.). The SER model is described as:

$$\mathbf{y} = \mathbf{Xb} + \mathbf{e}, \ \mathbf{e} \sim N_n(0, \sigma^2 I_n),$$
$$\mathbf{b} = \lambda\mathbf{c}, \ \mathbf{c} \sim Mult(1, \boldsymbol{\pi}), \ \lambda \sim N_1(0, \sigma_0^2). \tag{S.3}$$

Here, $\mathbf{y}$ is the n-vector of the centered response variable, $\mathbf{X} = (x_1, ..., x_p)$ is a scaled matrix containing n observations of p explanatory variables, $\mathbf{b}$ is the p-vector of regression coefficients which can be decomposed as the product of a scalar λ and indicator variable $\mathbf{c} = (c_1, ..., c_p)^T \in \{0, 1\}^p$, $\boldsymbol{\pi} = (\pi_1, ..., \pi_p)^T$ gives the prior probability that each variable is the effect variable, σ^2 and σ_0^2 are the hyperparameters for the residual variance and prior variance of the non-zero effect.

Under the SER model (S.2), there exists only one non-zero element in the coefficient vector $\mathbf{b}$, determined by the indicator variable $\mathbf{c}$. With fixed hyperparameters σ^2 and σ_0^2, the posterior distribution of $\mathbf{b} = \lambda\mathbf{c}$ can be computed as:

$$\mathbf{c}|\mathbf{X}, \mathbf{y}, \sigma^2, \sigma_0^2 \sim Mult(1, \boldsymbol{\alpha}),$$
$$\lambda|\mathbf{X}, \mathbf{y}, \sigma^2, \sigma_0^2, c_j = 1 \sim N_1(\mu_{1j}, \sigma_{1j}^2), \tag{S.4}$$

where $\boldsymbol{\alpha} = (\alpha_1, ..., \alpha_p)^T$ is the vector of PIPs, which can be computed with Bayes factors:

$$\alpha_j = Pr(c_j = 1|\mathbf{X}, \mathbf{y}, \sigma^2, \sigma_0^2) = \frac{\pi_j BF(x_j, \mathbf{y}; \sigma^2, \sigma_0^2)}{\sum_{j'=1}^{p} \pi_{j'} BF(x_{j'}, \mathbf{y}; \sigma^2, \sigma_0^2)}. \tag{S.5}$$

Suppose $\boldsymbol{\mu_1} = (\mu_{11}, ..., \mu_{1p})^T$, $\boldsymbol{\sigma_1^2} = (\sigma_{11}^2, ..., \sigma_{1p}^2)^T$, we can write the SER model as:

$$SER(\mathbf{X}, \mathbf{y}, \sigma^2, \sigma_0^2) := (\boldsymbol{\alpha}, \boldsymbol{\mu_1}, \boldsymbol{\sigma_1^2}). \tag{S.6}$$

Posterior inclusion probabilities for the single eQTL regression model:

In this section, we demonstrate that under the assumption that the distribution of phenotype given the gene expression level and genotype data is equivalent to the distribution of phenotype only given genotype data, the PIPs estimated by SuSiE[2] are exactly the posterior probabilities for variants to be causal given phenotype and gene expression level data. We first consider the following single eQTL regression (SEQR) model:

$$\mathbf{y} = \beta \mathbf{g} + \mathbf{e_1}, \quad \mathbf{e}_1 \sim N_n(0, \sigma_1^2 I_n), \tag{S.7}$$

$$\mathbf{g} = \mathbf{X}\mathbf{b} + \mathbf{e_2}, \quad \mathbf{e}_2 \sim N_n(0, \sigma_2^2 I_n),$$

$$\mathbf{b} = \lambda \mathbf{c},\ \mathbf{c} \sim Mult(1, \boldsymbol{\pi}),\ \lambda \sim N_1(0, \sigma_{20}^2), \tag{S.8}$$

where $\mathbf{y}$ is the n-vector of the centered phenotype, $\mathbf{g}$ is the n-vector of the centered gene expression level, $\mathbf{X}$ is the scaled genotype matrix of p SNPs, β is a fixed coefficient, $\mathbf{b}$ is defined in the same way as in the SER model.

The SEQR model can be considered as a two-layer model, where the second layer (S.8) can be considered as a SER model with the gene expression level $\mathbf{g}$ as the response variable. Assume a non-informative prior for each SNP to be causal in model (S.8), i.e., $\boldsymbol{\pi} = (1/p, ..., 1/p)^T$, then based on (S.5) in Property 2, the SuSiE[2] prior for SNP j to be causal is:

$$PIP_j^e = Pr(c_j = 1|\mathbf{X}, \mathbf{g}, \sigma_2^2, \sigma_{20}^2) = \frac{BF(x_j, \mathbf{g}; \sigma_2^2, \sigma_{20}^2)}{\sum_{j'=1}^{p} BF(x_{j'}, \mathbf{g}; \sigma_2^2, \sigma_{20}^2)}. \tag{S.9}$$

Suppose phenotype $\mathbf{y}$ is based on cohort one with n_1 samples, and gene expression level $\mathbf{g}$ is based on cohort two with n_2 samples. The genotype matrices of these two cohorts for p SNPs are $\mathbf{X}_1 = (\mathbf{x}_{11}, ..., \mathbf{x}_{1p})$ and $\mathbf{X}_2 = (\mathbf{x}_{21}, ..., \mathbf{x}_{2p})$, respectively.

Denote the parameters as $\phi = (\sigma_1^2, \sigma_2^2, \sigma_{20}^2, \beta)$, we can formulate the required assumption introduced in the first paragraph of this subsection as:

$$p(\mathbf{y}|\mathbf{g}, \mathbf{X}_1, \mathbf{X}_2, c_j = 1, \phi) = p(\mathbf{y}|\mathbf{X}_1, c_j = 1, \phi), \tag{S.10}$$

that is, the distribution of phenotype given the gene expression level and genotype data is equivalent to the distribution of phenotype given genotype data of cohort one.

Under this assumption, we can derive that:

$$\begin{aligned}
&Pr(\mathbf{y}, \mathbf{g}|\mathbf{X}_1, \mathbf{X}_2, c_j = 1, \phi) \\
=&\frac{Pr(\mathbf{y}, \mathbf{g}, \mathbf{X}_1, \mathbf{X}_2, c_j = 1, \phi)}{Pr(\mathbf{g}, \mathbf{X}_1, \mathbf{X}_2, c_j = 1, \phi)} \cdot \frac{Pr(\mathbf{g}, \mathbf{X}_1, \mathbf{X}_2, c_j = 1, \phi)}{Pr(\mathbf{X}_1, \mathbf{X}_2, c_j = 1, \phi)} \\
=&Pr(\mathbf{y}|\mathbf{g}, \mathbf{X}_1, \mathbf{X}_2, c_j = 1, \phi) \cdot Pr(\mathbf{g}|\mathbf{X}_1, \mathbf{X}_2, c_j = 1, \phi) \\
=&Pr(\mathbf{y}|\mathbf{X}_1, c_j = 1, \phi) \cdot Pr(\mathbf{g}|\mathbf{X}_2, c_j = 1, \phi).
\end{aligned} \tag{S.11}$$

It is worth mentioning that when the two cohorts are independent, which is usually the case in real analyses, this assumption is definitely valid. However, even when $\mathbf{y}$ and $\mathbf{g}$ are from the same cohort, we can still derive (S.11) based on this assumption.

In the SEQR model, the phenotype is effected by genotype only through the gene expression level, thus the causal SNP of gene expression level is also the only SNP that affects the phenotype. We can rewrite the SEQR model as the following two SER models between $\mathbf{y}, \mathbf{g}$ and genotype matrices $\mathbf{X}_1$ and $\mathbf{X}_2$, respectively:

$$\mathbf{y} = \beta(\mathbf{X}_1\mathbf{b} + \mathbf{e_2}) + \mathbf{e_1}, \quad \mathbf{e}_1 \sim N_n(0, \sigma_1^2 I_n), \tag{S.12}$$

$$\begin{aligned} \mathbf{g} &= \mathbf{X}_2\mathbf{b} + \mathbf{e_2}, \quad \mathbf{e}_2 \sim N_n(0, \sigma_2^2 I_n), \\ \mathbf{b} &= \lambda\mathbf{c},\ \mathbf{c} \sim Mult(1, \boldsymbol{\pi}),\ \lambda \sim N_1(0, \sigma_{20}^2). \end{aligned} \tag{S.13}$$

With the decomposition (S.11), under a non-informative prior $Pr(c_j = 1) = \frac{1}{p}$, the posterior probability for SNP j to be causal for phenotype $\mathbf{y}$ is:

$$\begin{aligned} &Pr(c_j = 1|\mathbf{y}, \mathbf{g}, \mathbf{X}_1, \mathbf{X}_2, \phi) \\ =&\frac{Pr(\mathbf{y}, \mathbf{g}|\mathbf{X}_1, \mathbf{X}_2, c_j = 1, \phi)Pr(\mathbf{X}_1, \mathbf{X}_2, \phi)Pr(c_j = 1)}{\sum_{j'=1}^{p} Pr(\mathbf{y}, \mathbf{g}|\mathbf{X}_1, \mathbf{X}_2, c_{j'} = 1, \phi)Pr(\mathbf{X}_1, \mathbf{X}_2, \phi)Pr(c_{j'} = 1)} \\ =&\frac{Pr(\mathbf{y}, \mathbf{g}|\mathbf{X}_1, \mathbf{X}_2, c_j = 1, \phi)}{\sum_{j'=1}^{p} Pr(\mathbf{y}, \mathbf{g}|\mathbf{X}_1, \mathbf{X}_2, c_{j'} = 1, \phi)} \\ =&\frac{Pr(\mathbf{y}|\mathbf{X}_1, c_j = 1, \phi) \cdot Pr(\mathbf{g}|\mathbf{X}_2, c_j = 1, \phi)}{\sum_{j'=1}^{p} Pr(\mathbf{y}|\mathbf{X}_1, c_{j'} = 1, \phi) \cdot Pr(\mathbf{g}|\mathbf{X}_2, c_{j'} = 1, \phi)}. \end{aligned} \tag{S.14}$$

Suppose $c_j = 1$, then SNP j is the only variant with non-zero coefficient for $\mathbf{g}$ and $\mathbf{y}$. Therefore, $\mathbf{y}$ is decided by the following simple linear regression model:

$$\begin{aligned} &\mathbf{y} = \beta(\mathbf{x}_{1j}\lambda + \mathbf{e}_2) + \mathbf{e}_1 = \mathbf{x}_{1j}\beta' + \mathbf{e}_1', \\ &\beta' = \beta \cdot \lambda \sim N_1(0, \beta^2\sigma_{20}^2),\ \mathbf{e}_1' = \beta\mathbf{e}_2 + \mathbf{e}_1 \sim N_n(0, \sigma^2 I_n). \end{aligned} \tag{S.15}$$

where $\sigma^2 := \beta^2\sigma_2^2 + \sigma_1^2$, then based on (S.2) in Property 1, we have

$$\begin{aligned} Pr(\mathbf{y}|\mathbf{X}_1, c_j = 1, \phi) =&BF(\mathbf{x}_{1j}, \mathbf{y}; \sigma^2, \beta^2\sigma_{20}^2) \cdot Pr(\mathbf{y}|\mathbf{x}_{1j}, \beta' = 0, \phi) \\ =&BF(\mathbf{x}_{1j}, \mathbf{y}; \sigma^2, \beta^2\sigma_{20}^2) \cdot \left(\frac{1}{\sqrt{2\pi\sigma^2}}\right)^n exp\{-\frac{\mathbf{y^T y}}{2\sigma^2}\}. \end{aligned} \tag{S.16}$$

Similarly, consider the gene expression level, we have:

$$\begin{aligned} Pr(\mathbf{g}|\mathbf{X}_2, c_j = 1, \phi) =&BF(\mathbf{x}_{2j}, \mathbf{g}; \sigma_2^2, \sigma_{20}^2) \cdot Pr(\mathbf{g}|\mathbf{x}_{2j}, \lambda = 0, \phi) \\ =&BF(\mathbf{x}_{2j}, \mathbf{g}; \sigma_2^2, \sigma_{20}^2) \cdot \left(\frac{1}{\sqrt{2\pi\sigma_2^2}}\right)^n exp\{-\frac{\mathbf{g^T g}}{2\sigma_2^2}\}. \end{aligned} \tag{S.17}$$

Therefore, with (S.16) and (S.17), we have

$$\begin{aligned} &Pr(c_j = 1|\mathbf{y}, \mathbf{g}, \mathbf{X}_1, \mathbf{X}_2, \phi) \\ =&\frac{Pr(\mathbf{y}|\mathbf{X}_1, c_j = 1, \phi) \cdot Pr(\mathbf{g}|\mathbf{X}_2, c_j = 1, \phi)}{\sum_{j'=1}^{p} Pr(\mathbf{y}|\mathbf{X}_1, c_{j'} = 1, \phi) \cdot Pr(\mathbf{g}|\mathbf{X}_2, c_{j'} = 1, \phi)} \\ =&\frac{BF(\mathbf{x}_{1j}, \mathbf{y}; \sigma^2, \beta^2\sigma_{20}^2) \cdot BF(\mathbf{x}_{2j}, \mathbf{g}; \sigma_2^2, \sigma_{20}^2)}{\sum_{j'=1}^{p} BF(\mathbf{x}_{1j'}, \mathbf{y}; \sigma^2, \beta^2\sigma_{20}^2) \cdot BF(\mathbf{x}_{2j'}, \mathbf{g}; \sigma_2^2, \sigma_{20}^2)} \\ =&\frac{BF(\mathbf{x}_{1j}, \mathbf{y}; \sigma^2, \beta^2\sigma_{20}^2) \cdot PIP_j^e}{\sum_{j'=1}^{p} BF(\mathbf{x}_{1j'}, \mathbf{y}; \sigma^2, \beta^2\sigma_{20}^2) \cdot PIP_{j'}^e}, \end{aligned} \tag{S.18}$$

where PIP_j^e is the SuSiE^2 prior for SNP j to be causal, as defined in (S.9).

Therefore, under the assumption that the distribution of phenotype given the gene expression level and genotype data is equivalent to the distribution of phenotype given genotype data (S.10), we conclude that the estimated PIP given phenotype and gene expression level $Pr(c_j = 1|\mathbf{y}, \mathbf{g}, \mathbf{X}_1, \mathbf{X}_2, \phi)$ is equivalent to the SuSiE estimator under the SER model (S.12) when we choose the prior to be the PIP from the eQTL-based SuSiE model, which is exactly the SuSiE^2 estimator.

2 Details of the methods compared in simulation.

In this section, we describe how we use all the compared fine-mapping methods in simulations.

SuSiE: We ran SuSiE in all simulations by calling the function `susie_rss` from the `susieR` package (version 0.12.40) [1,2], which performs SuSiE regression with summary statistics and the LD matrix. For simulation scenario (a) and (b), we set the maximum number of non-zero effects (L) to 10. Throughout our simulations, we set the minimum absolute correlation allowed in a credible set to be the default 0.5, and asked the function to estimate the residual variance (`estimate_residual_variance=TRUE`), which is recommended by SuSiE (the same for SuSiE^2 and mvSuSiE).

SuSiE^2: We ran SuSiE^2 with `susie_rss` in three steps. In step 1, we apply SuSiE to perform cis-eQTL fine-mapping for all risk genes in the simulated locus, with the number of non-zero effects set to be 2 for each risk gene region. In step 2, we integrate eQTL information from multiple risk genes by modifying the prior inclusion probability within gene regions to be PIPs from eQTL-based SuSiE for the corresponding gene. The prior probabilities of the remaining SNPs are set to be $\frac{max(1,d)}{p}$, where d is the total number of causal SNPs not in gene regions. In step 3, we conduct SuSiE regression for trait of interest with prior inclusion probabilities obtained from step 2. For simulation scenario (a) and (b), we set L = 10. For simulation scenario (c), we set L = 30. We set the minimum absolute correlation to be 0.5.

mvSuSiE: We conducted multi-trait fine-mapping with mvSuSiE using the `mvsusie_rss` function from the `mvsusieR` R package (version 0.1.5) [3]. We calculate a $p \times (R + 1)$ matrix of z-scores for trait of interest and gene expression levels, where R is the total number of risk genes. We used the `create_mixture_prior` function from the `mvSuSiE` package to create the canonical prior. For simulation scenario (a) and (b), we set L = 10. We say a credible set is significant for the phenotype if the average local false sign rate (lfsr) in the credible set is less than 0.05. We ran `mvsusie_rss` with the following setting: `estimate_prior_variance = TRUE, min_abs_corr = 0.5, estimate_prior_method = "EM"`.

fastPAINTOR: We conducted multi-trait fine-mapping with fastPAINTOR [4] using the `PAINTOR v3.1` package. The input files for the function `PAINTOR` are locus files that contains the z-scores for the phenotype and gene expression levels, the LD matrix files for all traits, and the annotation matrix file. We ran fastPAINTOR without annotations by creating a "dummy" annotation file with all elements to be 1. We conducted the approximate inference using a Gibbs sampling algorithm (`-mcmc`). For scenario (a) and (b), we specified the number of causals SNPs to 10.

The output of `PAINTOR` is a column of posterior probabilities for each SNP to be causal for both phenotype and gene expression levels. Therefore, we constructed credible sets based on posterior probabilities and the LD matrix, using a similar method to [5],

but fixed the minimum absolute correlation for SNPs from the same credible set at 0.5.

flashfm: We ran flashfm using the `FLASHFMwithFINEMAP` function from R package `flashfm` (version 0.0.0.9000) [6]. This function internally applied FINEMAP (we used FINEMAP version v1.4.2) [7] to perform multi-trait fine-mapping. The inputs to `FLASHFMwithFINEMAP` are a list of summary statistics, a SNP correlation matrix, a vector of reference allele frequency, a vector of trait means, and a vector of sample sizes for traits. All other settings were kept at their defaults. Since both the trait of interests and the genotype matrix were standardized, we set the trait means to zero, and replaced the MAFs with a constant value as these MAFs were used to obtain the covariance matrix from the LD matrix. Since at most 5 traits can be used with the `FLASHFMwithFINEMAP` function, we can perform flashfm on simulation scenario (a) and (b).

3 URLs.

susieR: `https://github.com/stephenslab/susieR`
mvSuSiE: `https://github.com/stephenslab/mvsusieR`
fastPAINTOR: `https://github.com/gkichaev/PAINTOR_V3.0`
flashfm: `https://jennasimit.github.io/flashfm/`
FINEMAP: `http://www.christianbenner.com`
UK Biobank genotype data: `https://www.ukbiobank.ac.uk/`
UK Biobank BMI GWAS:
`https://biobank.ctsu.ox.ac.uk/crystal/field.cgi?id=21001`
`https://www.nealelab.is/uk-biobank`
GTEx: `https://gtexportal.org/home/`; dbGaP Accession phs000424.v8.p2
The Alzheimer's Disease summary statistics:
`https://ctg.cncr.nl/software/summary_statistics`
ROSMAP Gene Expression data:
`https://www.synapse.org/#!Synapse:syn17008934`
Validation data for AD risk genes: `https://doi.org/10.1038/s41588-020-00721-x`

4 Iterative Bayesian stepwise selection (IBSS) algorithm.

Algorithm 1 IBSS

Require: data $\mathbf{X}, \mathbf{y}$, number of effects K, hyperparameters $\sigma^2, \boldsymbol{\sigma_0^2}$
Initialize posterior means $\bar{\mathbf{b}}_\mathbf{k} = 0$, $k = 1, .., K$
repeat
  for `k in 1,...,K` **do**
    $\bar{\mathbf{r}}_\mathbf{k} \leftarrow \mathbf{y} - \mathbf{X} \sum_{k' \neq k} \bar{\mathbf{b}}_{\mathbf{k'}}$ ▷ expected residuals without kth single effect
    $(\boldsymbol{\alpha_k}, \boldsymbol{\mu_{1k}}, \boldsymbol{\sigma_k^2}) \leftarrow SER(\mathbf{X}, \bar{\mathbf{r}}_\mathbf{k}, \sigma^2, \sigma_{0k}^2)$
    $\bar{\mathbf{b}}_\mathbf{k} \leftarrow \boldsymbol{\alpha_k} \cdot \boldsymbol{\mu_{1k}}$ ▷ $\cdot$ denotes elementwise multiplication
  end for
until convergence **return** $\boldsymbol{\alpha_k}, \boldsymbol{\mu_{1k}}, \boldsymbol{\sigma_k^2}$

References

1. Wang G, Sarkar A, Carbonetto P, Stephens M. A simple new approach to variable selection in regression, with application to genetic fine mapping. Journal of the Royal Statistical Society Series B: Statistical Methodology. 2020;82(5):1273–1300.

2. Zou Y, Carbonetto P, Wang G, Stephens M. Fine-mapping from summary data with the "Sum of Single Effects" model. PLoS Genetics. 2022;18(7):e1010299.

3. Zou Y, Carbonetto P, Xie D, Wang G, Stephens M. Fast and flexible joint fine-mapping of multiple traits via the Sum of Single Effects model. bioRxiv. 2023; p. 2023–04.

4. Kichaev G, Roytman M, Johnson R, Eskin E, Lindstroem S, Kraft P, et al. Improved methods for multi-trait fine mapping of pleiotropic risk loci. Bioinformatics. 2017;33(2):248–255.

5. Yang Z, Wang C, Liu L, Khan A, Lee A, Vardarajan B, et al. CARMA is a new Bayesian model for fine-mapping in genome-wide association meta-analyses. Nature Genetics. 2023; p. 1–9.

6. Hernández N, Soenksen J, Newcombe P, Sandhu M, Barroso I, Wallace C, et al. The flashfm approach for fine-mapping multiple quantitative traits. Nature Communications. 2021;12(1):6147.

7. Benner C, Spencer CC, Havulinna AS, Salomaa V, Ripatti S, Pirinen M. FINEMAP: efficient variable selection using summary data from genome-wide association studies. Bioinformatics. 2016;32(10):1493–1501.
