# Supplementary figures and images for: Integration of expression QTLs with fine mapping via SuSiE
Source: PLoS Genet. 2024 Jan 25;20(1):e1010929. doi: 10.1371/journal.pgen.1010929 (PMC10846745; doi:10.1371/journal.pgen.1010929)

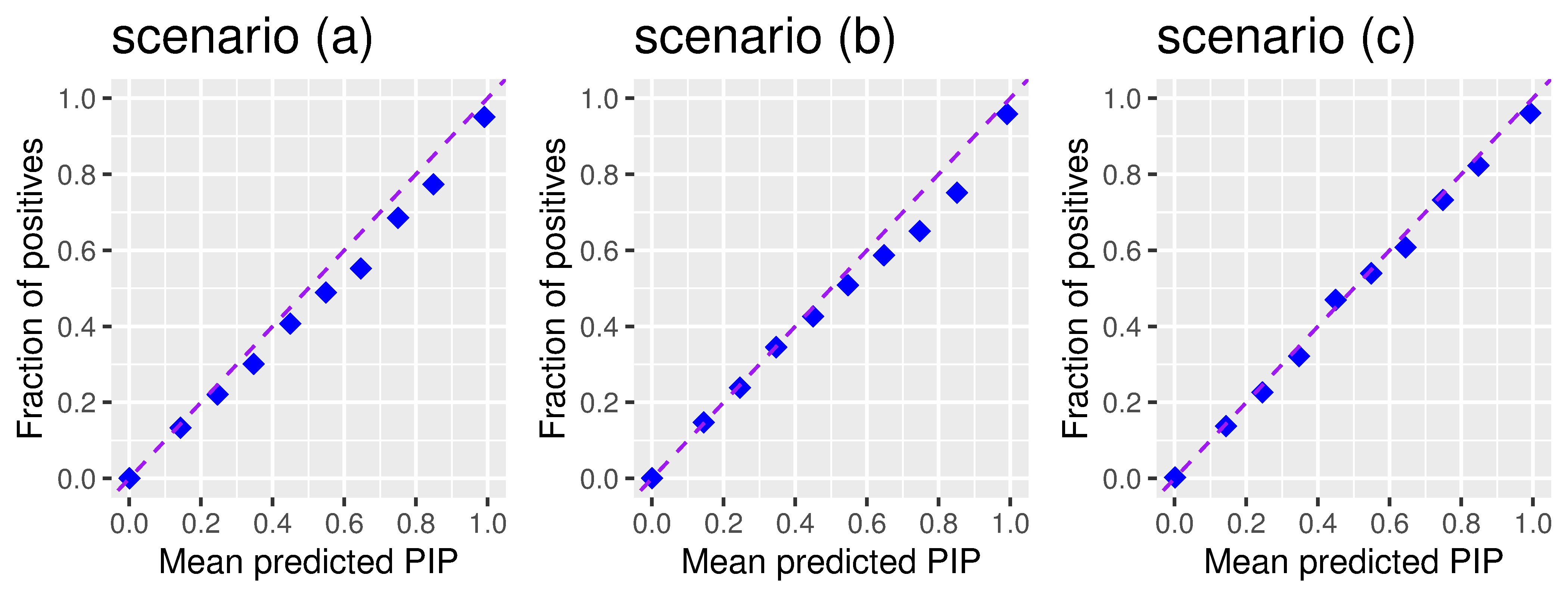

Supplement: S1 Fig — In each scenario, we repeated the simulation for 1,000 times and grouped the SNPs into 10 evenly spaced bins from 0 to 1 according to their PIP estimated by SuSiE2. The x-axis of this calibration figure is the average of predicted PIP for each bin, and the y-axis is the fraction of causal SNPs in each bin. The dashed line corresponds to the y = x diagonal line. A well-calibrated method should produce points close to the diagonal line. (TIF) [file pgen.1010929.s003.tif]

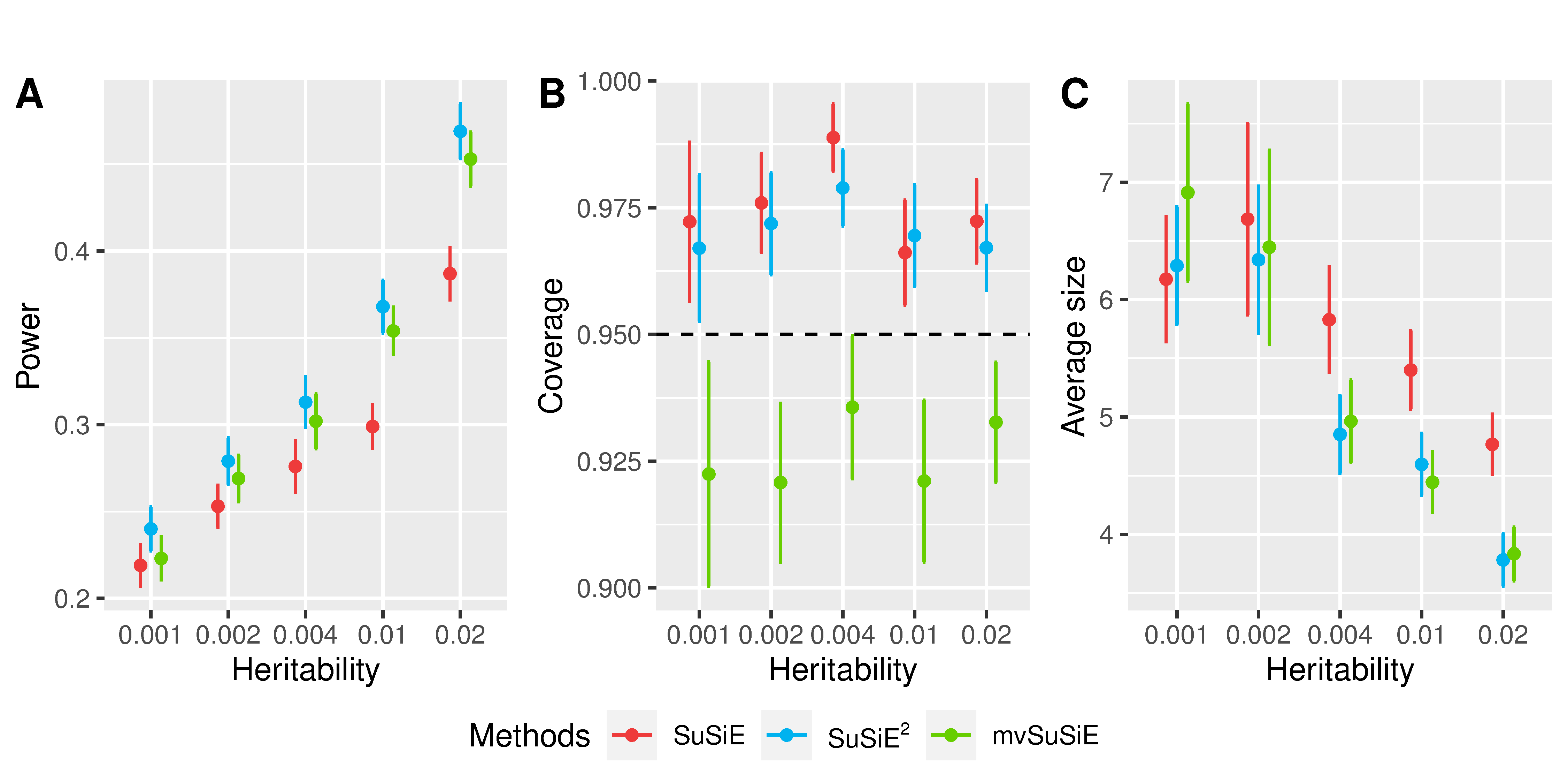

Supplement: S2 Fig — We compare the 95% credible sets from SuSiE, SuSiE2, and mvSuSiE under scenario (b). The heritability of direct effect for phenotype (1-σt2) is fixed at 0.1. The heritability for the eQTL study (1-σel2) increased from 0.001 to 0.02. Panel A evaluates the power of detecting causal SNPs in at least one credible set. Panel B evaluates the coverage of credible sets, with the black dashed line corresponding to the 95% level. Panel C evaluates the average size of credible sets. (TIF) [file pgen.1010929.s004.tif]

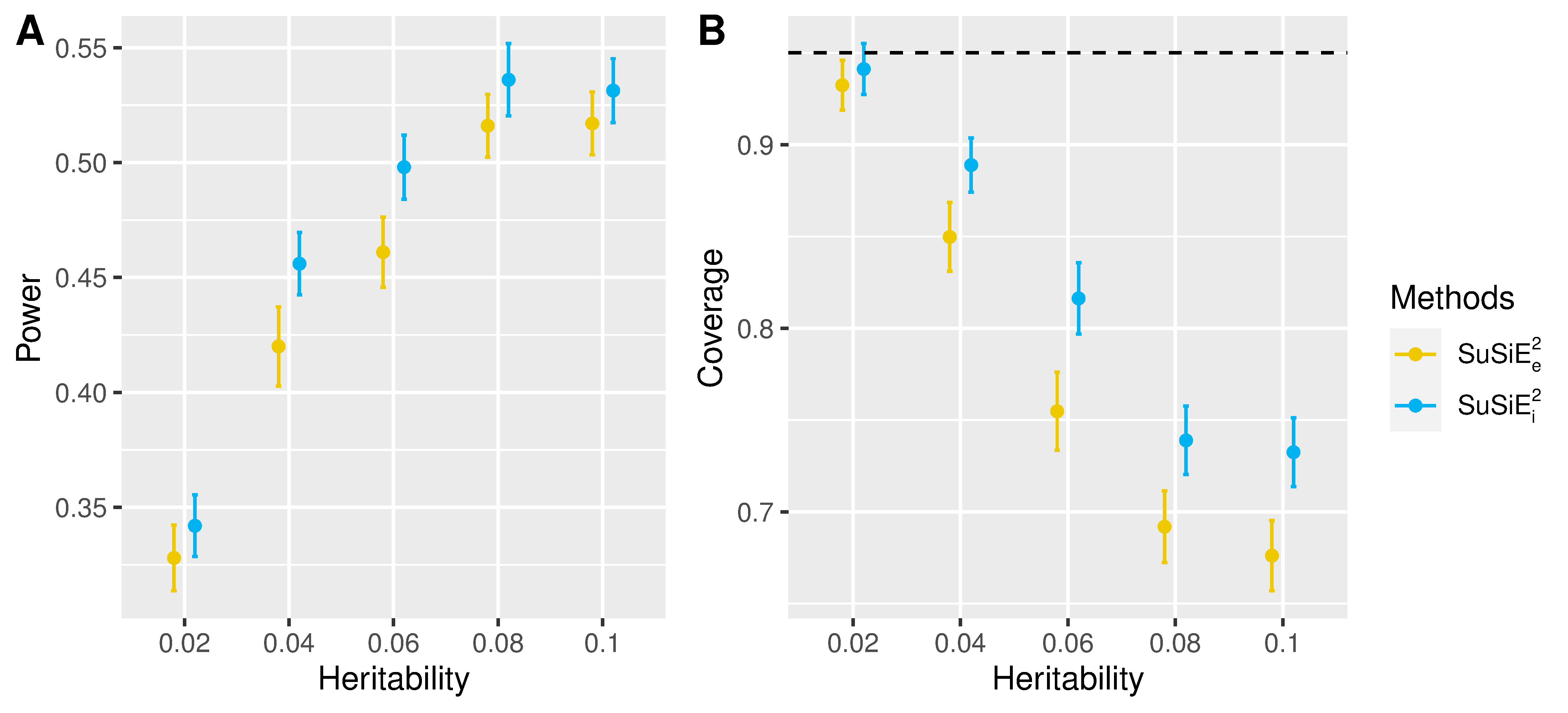

Supplement: S3 Fig — We compare the 95% credible sets from SuSiE2 when using either the in-sample LD matrix (SuSiEi2) or the LD matrix from 1KG panel (SuSiEe2) in the eQTL-based SuSiE. For each combination of method and heritability, we show the mean value and the standard error from 150 repetitions. Panel A evaluates the power of detecting causal SNPs in at least one credible set. Panel B evaluates the coverage of credible sets, with the black dashed line corresponding to the 95% level. For the second step of SuSiE2 (fine-mapping for the trait of interest), we always used the LD matrix from the 1KG reference panel. (TIF) [file pgen.1010929.s005.tif]

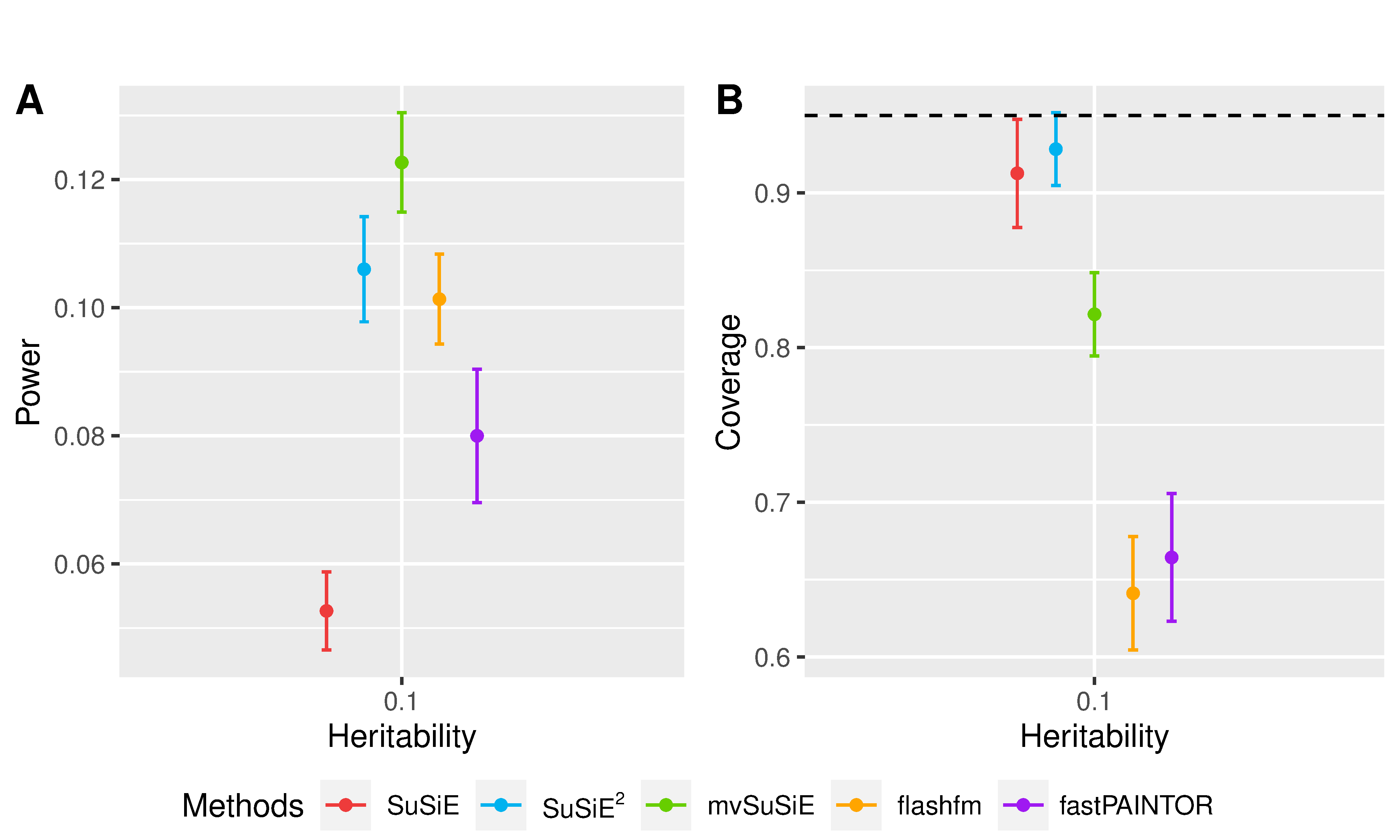

Supplement: S4 Fig — We compare the 95% credible sets from five fine-mapping methods (SuSiE, SuSiE2, mvSuSiE, flashfm, fastPAINTOR) under scenario (b). The total heritability was fixed at 0.1. For each combination of method and heritability, we show the mean value and the empirical standard error from 150 repetitions. Panel A evaluates the power of detecting causal SNPs in at least one credible set. Panel B evaluates the coverage of credible sets, with the black dashed line corresponding to the 95% level. Phenotype and gene expression levels were simulated based on the genotypes of 5,000 SNPs from 503 1KG samples. The reference panel consisted of 10,000 UKBB samples. (TIF) [file pgen.1010929.s006.tif]
